# Preparation of polysaccharide composite films using cyclodextrin-conjugated chitosan for sustained release of hydrophobic drugs

**DOI:** 10.1039/d5ra07595e

**Published:** 2025-11-24

**Authors:** Takuya Sagawa, Aoi Kashiwabara, Mineo Hashizume

**Affiliations:** a Department of Industrial Chemistry, Faculty of Engineering, Tokyo University of Science 6-3-1 Niijuku, Katsushika-ku Tokyo 125-8585 Japan t_sagawa@ci.tus.ac.jp mhashizu@ci.tus.ac.jp; b Graduate School of Engineering, Tokyo University of Science 6-3-1 Niijuku, Katsushika-ku Tokyo 125-8585 Japan

## Abstract

Cyclodextrin-conjugated polysaccharides are often used as functional materials for biological applications, such as drug carriers, because the hydrophobic cavity of the cyclodextrin moiety can encapsulate molecules. Herein, β-cyclodextrin (β-CD)–conjugated chitosan (CD–CHI) for loading and sustained-release of low-molecular-weight hydrophobic drugs was synthesized, and polysaccharide composite films containing β-CD units were prepared from polyion complexes consisting of chondroitin sulfate C and CD–CHI by hot press techniques. β-CD units of the obtained CD–CHI were modified by 9.2% of the amino groups in CHI units. The synthesized CD–CHI was used as a raw material for polysaccharide composite films. The mechanical strength and swelling ratio of the obtained films were comparable to those of films without β-CD. Furthermore, thiabendazole (TBZ) was loaded into the polysaccharide composite films, and it was suggested that the loaded TBZ formed inclusion complexes with the β-CD units in CD–CHI. The loaded TBZ showed sustained-release ability, and the release mechanism from the films was analyzed and described using two kinetic models. Based on these results, polysaccharide composite films using CD–CHI are expected to be used as sustained-release carriers for hydrophobic drugs.

## Abstract 2

Polysaccharide composite films were prepared from polyion complexes composed of chondroitin sulfate C and β-cyclodextrin-conjugated chitosan by hot press techniques. The film can be used as a hydrophobic drug carrier with a tunable loading amount.

## Graphical abstract

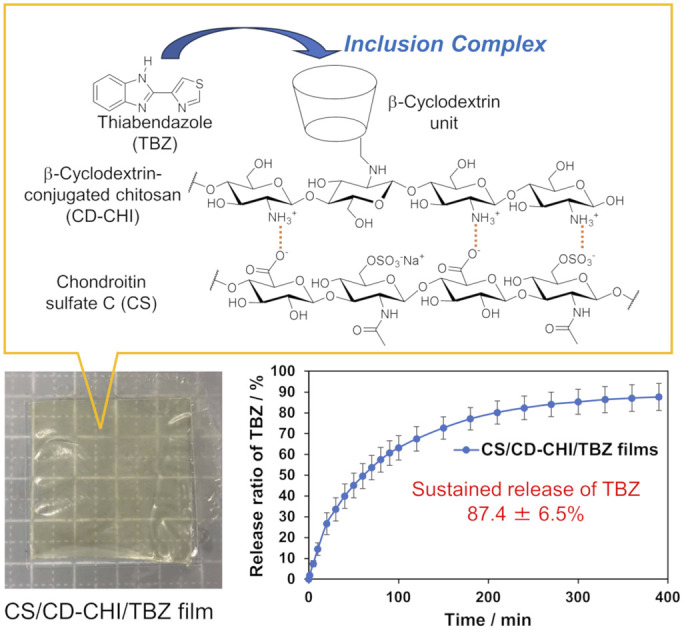

## 1. Introduction

From a viewpoint of the Sustainable Development Goals (SDGs), the fabrication of structural materials from biomass is expected to reduce environmental impact. In particular, natural polysaccharides are abundant and show biocompatibility, biodegradability, and non-toxicity. Therefore, these have often been used for biomaterials such as drug carriers.^1–5^

**Fig. 1:**
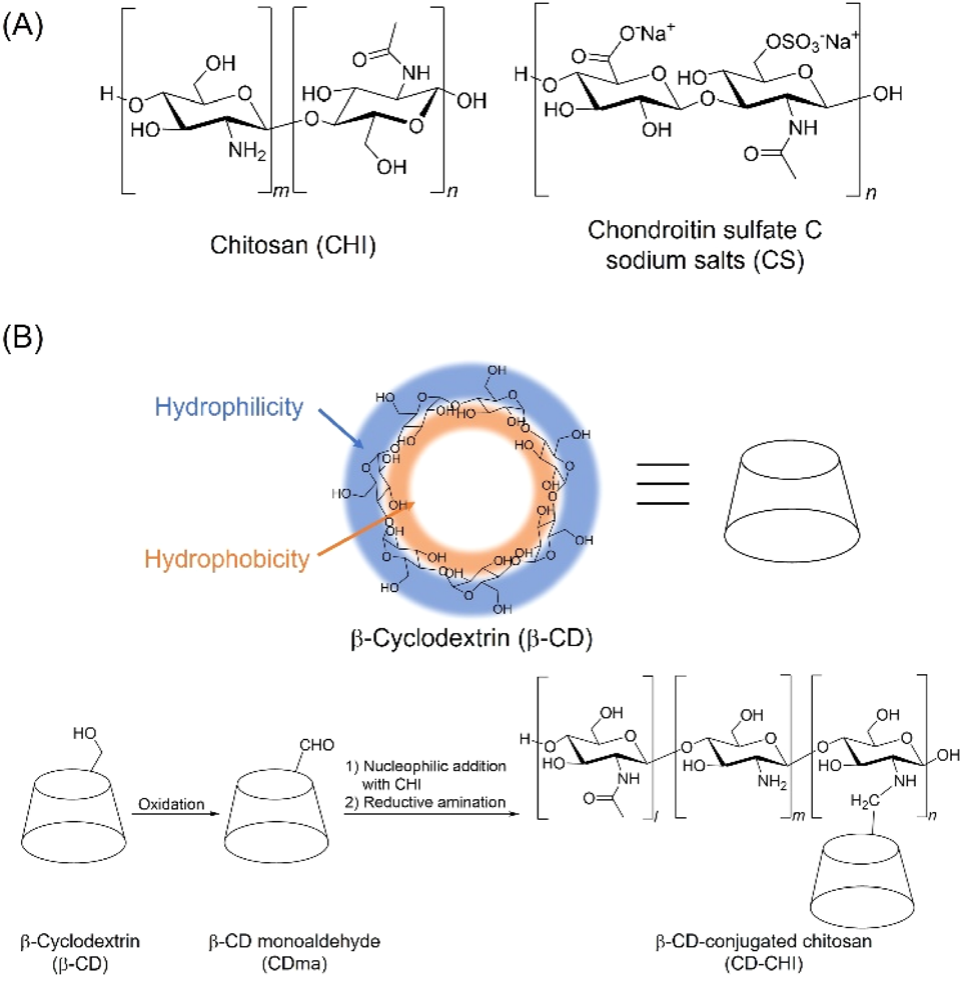
(A) Structure of chitosan (CHI) and chondroitin sulfate C sodium salts (CS). (B) Synthetic scheme of CD–CHI from β-CD.

Polysaccharides are classified as water-soluble and insoluble, and water-soluble polysaccharides show higher moldability than water-insoluble polysaccharides such as cellulose and chitin.^6^ Among water-soluble polysaccharides, chitosan (CHI) and chondroitin sulfate C (CS) form polyions in aqueous solutions with appropriate pH (Fig. 1A). Notably, CHI exhibits biological properties such as biocompatibility and antibacterial properties. Therefore, it can be used as structural materials for the preservation of waterlogged archaeological wood,^7^ as gel systems for the removal of pollutants,^8^ and as biomaterials such as wound dressings and drug carriers.^9^ Furthermore, they can electrostatically interact with oppositely charged inorganic ions, proteins, and polysaccharides.^10–12^ To date, the use of cross-linking agents or the formation of polyion complexes (PICs) has been reported to enhance the durability of structural materials composed of water-soluble polysaccharides.^13–18^ In particular, the formation of PICs has received attention because the intrinsic chemical properties of polysaccharides are maintained.

Among structural materials, film-like materials are utilized as wound dressings, drug carriers, and cell scaffolds.^19–23^ Recently, film materials made of PICs have been fabricated by layer-by-layer (LbL) assembling techniques.^24–26^ However, most of these films had a thickness of only a few tens of nanometers and did not show free-standing properties. To obtain LbL-type films with micrometer thicknesses, multiple repetitions of assembly and a prolonged processing time were required.^26^ On the other hand, we have reported the fabrication of polysaccharide composite films from PICs obtained by mixing CS and CHI using hot press techniques (denoted as CS/CHI films).^27–36^ These films are insoluble and swell in water and buffer solutions at any pH. Therefore, these films can be used as drug carriers with sustained drug release ability. Actually, molecular loading and release abilities of these films for cationic molecules, such as methylene blue and α,β,γ,δ-tetrakis(1-methylpyridinium-4-yl)porphyrin *p*-toluenesulfonate, and anionic molecules, such as fluorescein, have been reported.^29,34,35^ These molecules can interact with polysaccharides by electrostatic and hydrogen bonding interactions. Meanwhile, we also attempted the loading of thiabendazole (TBZ), a model hydrophobic drug known as an anthelmintic and antifungal drug,^36^ into the CS/CHI films; however, only a small amount of TBZ was loaded due to its weak interactions with polysaccharides.^37^ Many drugs with low molecular weights in practical use show hydrophobicity. Hence, CS/CHI films capable of loading large amounts of hydrophobic drugs can be expected to expand the range of applications as DDS materials.

In terms of the above-mentioned improvement in hydrophobic drug loading ability, the formation of inclusion complexes with cyclodextrins (CDs) is one of the methods for solubilizing low molecular weight hydrophobic drugs.^38–40^ CDs have a hydrophobic cavity that forms inclusion complexes with guest hydrophobic molecules, allowing for the sustained release of the encapsulated hydrophobic molecules. Thus, CDs are often used as drug carriers.^41–43^ Recently, CD-grafted polysaccharides have been reported to improve the solubilization, stabilization, and transport of hydrophobic drugs.^44–46^ For example, CD-grafted CHI comprising CHI and β-CD can encapsulate hydrophobic drugs and sustain drug release.^47–49^ Thus, polysaccharide composite films prepared using CD-grafted CHI as a raw material are expected to serve as drug carriers for TBZ, thereby improving its loading and release ability. Furthermore, by changing the number of CD units in CD-grafted CHI, the loading amount of drugs could be adjusted. Moreover, we have reported that the release behaviour of drugs in CS/CHI films depends on the pH and salt strength of the solution.^29^ Hence, the release behaviour of drugs from polysaccharide composite films using CD-grafted CHI can also be affected by these conditions, suggesting that the films can be utilized as stimuli-responsive materials. However, there have been no reports to date on the preparation of polysaccharide composite films using modified polysaccharides such as CD-grafted CHI. Since the main driving force for the formation of PICs is electrostatic interaction, it is necessary to choose CD-grafted CHI, which maintains the proton affinity of its amino groups. In this study, to improve the loading and release ability of TBZ, polysaccharide composite films composed of CS and CD-grafted CHI were prepared from their PICs by hot-press techniques. The novelty of this study is that, for the first time, the films were prepared from PICs using a modified polysaccharide, CD-grafted CHI, and a hydrophobic drug was successfully loaded into and released from polysaccharide composite films, which has been difficult to achieve so far. CD-grafted CHI was prepared by the nucleophilic addition reaction of monoaldehyde-β-cyclodextrin (CDma) with CHI and subsequent reductive amination (denoted as CD–CHI, Fig. 1B). CD–CHI contains primary and secondary amines that can be protonated at an appropriate pH. Therefore, the number of cationic units does not change even after conjugation of the CD units. The obtained polysaccharide composite films (CS/CD–CHI films) were characterized, and their physical properties were evaluated. Furthermore, the drug loading and sustained release ability were evaluated using TBZ as a hydrophobic drug. This study aims to achieve the loading and sustained release of TBZ, which was difficult with conventional CD/CHI films, by using CS/CD–CHI films, and to clarify the drug release behaviour.

## 2. Experimental

### 2.1. Materials

β-Cyclodextrin (β-CD), Dess–Martin periodinane (DMP), 2-picoline borane, and thiabendazole (TBZ) were purchased from Tokyo Chemical Industry. Dimethylsulfoxide (DMSO, super dehydrate) was purchased from Fujifilm Wako Pure Chemical Corporation. Chondroitin sulfate C (CS, sodium salt, from shark cartilage, molecular weight (MW) *ca.* 20 000), chitosan (CHI, from crab shell, MW ≥ 100 000, deacetylation degree was approximately 86%), acetic acid, methanol, acetone, and other chemicals were obtained from Nacalai Tesque Inc. All chemicals were used as received. Distilled water and ultrapure water (18.2 MΩ cm) were prepared for the experiments (RFD210TA and RFU414BA, respectively; Advantec Toyo Kaisha, Ltd).

### 2.2. Synthesis of β-CD–conjugated chitosan (CD–CHI)

Firstly, CDma was synthesized by oxidation of the hydroxy group at position 6 of the glucose unit in β-CD using Dess–Martin periodinane.^50,51^ Then, CD–CHI was synthesized by nucleophilic addition to the formyl group in CDma with an amino group in CHI to form a Schiff base, and subsequent reductive amination (Fig. 1B).^50^ The details of the synthetic method for CDma and CD–CHI are described in the SI.

### 2.3. Preparation of polysaccharide composite films composed of CS and CD–CHI (CS/CD–CHI film)

**Fig. 2:**
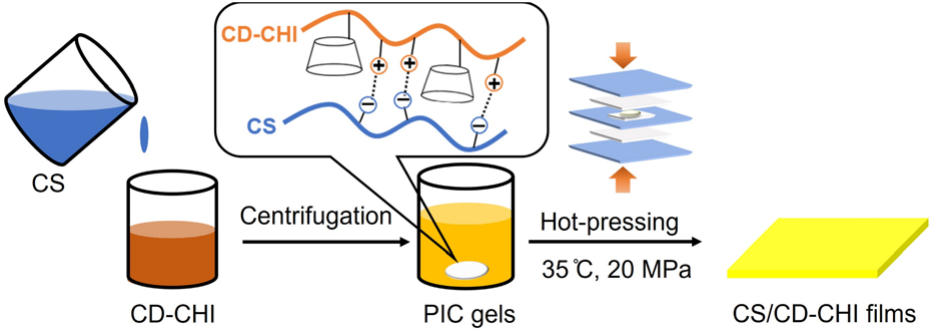
Schematic of the preparation of the CS/CD–CHI films.

Schematic illustration of the preparation of polysaccharide composite films using CD–CHI is shown in Fig. 2. The preparation of CS/CD–CHI films was carried out by a process described in our previous report with several modifications.^27,34^ CD–CHI (0.10 g) was dissolved in a 2.0 wt% acetic acid aqueous solution (10 mL) to obtain a 1.0 wt% CD–CHI solution, and CS (0.70 g) was dissolved in ultrapure water (35 mL) to obtain a 2.0 wt% CS solution. Then, the solution was added dropwise to a 1.0 wt% CD–CHI solution (7.5 mL, 25.6 µmol of CD units) until the insoluble PICs were completely formed. The sample was shaken in a vortex mixer (Vortex-Genie 2, Scientific Industries) at room temperature for 20 s and was centrifuged (6000 rpm, 5 min). After that, the supernatant was decanted, and ultrapure water (30 mL) was added to wash the gel. The mixture was centrifuged (6000 rpm, 5 min), and PIC gels composed of CS and CD–CHI were obtained. The gels were used for the subsequent film preparation without drying.

The obtained gels were sandwiched between polytetrafluoroethylene (PTFE, thickness: 100 µm) and polyethylene terephthalate (PET, thickness: 100 µm) sheets and placed on the stage of a hot press apparatus (H300-15, AS ONE Corp.) preheated to 35 or 120 °C. The gels were hot pressed at 20 MPa for 5 min at 35 or 120 °C. Excess water was removed using a wiper, and then the gels were folded into pieces of *ca.* 1.0 × 1.0 cm. The gels were further hot pressed at 20 MPa for 5 min at 35 °C. After that, the gels were folded again into pieces of *ca.* 1.0 × 1.0 cm. The gels were placed in the centre of a PET sheet; the centre of the film was a square cutout (4.0 × 4.0 cm), and the gels were further hot-pressed at 20 MPa for 5 min at 35 °C. After hot-pressing, the resulting films were dried in a desiccator and trimmed with cutter knives to obtain the CS/CD–CHI films. Numerous examinations revealed that such a procedure, including swelling and folding processes, was necessary to obtain homogenous films reproducibly.^24,25^ Furthermore, polysaccharide composite films composed of CS and CHI (CS/CHI films) were also prepared using the previous method with several modifications.^27,34^

### 2.4. Preparation of β-CD-loaded CS/CHI film (CD/CS/CHI film)

To evaluate the effect of covalent bonding between the CD unit and CHI, CS/CHI films loaded with unmodified β-CD (CD/CS/CHI films) were prepared. CHI (0.45 g) and β-CD (0.55 g) were dissolved in a 2.0% acetic acid aqueous solution to prepare a 1.0 wt% CD/CHI solution. The preparation of CS solution, PIC gels, and CD/CS/CHI films was similar to that described in Section 2.3. To calculate the amount of loaded β-CD in CD/CS/CHI films, the amount of β-CD in a supernatant and washing solution that was generated at the PIC gel preparation procedure was measured by high-performance liquid chromatography (HPLC) equipped with a refractive index detector. An SB-802.5M HQ column and an SB-803M HQ column were connected as size exclusion columns. The column temperature was 40 °C, and the mobile phase was 50 mM acetic acid with 3 M NaNO_3_ aqueous solution (flow rate: 0.5 mL min^−1^). The amounts of β-CD were calculated by an absolute calibration method (Fig. S1).

### 2.5. Preparation of TBZ-loaded CS/CD–CHI film (CS/CD–CHI/TBZ film)

1.0 wt% CD–CHI solution was prepared by the same method as described in Section 2.3. TBZ (0.0364 g) was dissolved in 5 mL of ethanol at 60 °C to prepare a 3.6 mM TBZ solution. The TBZ solution (1.0 mL, 36.2 µmol) and 1.0 wt% CD–CHI solution (7.5 mL, 25.6 µmol of CD units) were mixed, and the solution was stirred at 70 °C for 4 hours. Then, a part of ethanol was removed at 80 °C, and the mixture was cooled to room temperature to obtain the TBZ-loaded CD–CHI (CD–CHI/TBZ). CD–CHI/TBZ was characterized by ^1^H NMR measurements in 2.0% acetic acid-d_4_ solution. In a control experiment, the ^1^H NMR measurement of TBZ was also performed. Afterward, 2.0 mL of CS solution was added to the mixture, and the PICs and CS/CD–CHI/TBZ films were fabricated by a similar method as given in Section 2.3.

### 2.6. Preparation of TBZ-loaded CS/CHI film (CS/CHI/TBZ film)

TBZ (0.0364 g) was dissolved in ethanol (5 mL) at 60 °C to prepare a 3.6 mM TBZ solution. The TBZ solution (1.0 mL, 36.2 µmol) was added to a 1.0 wt% CHI solution, and the mixture was then mixed with a 2.0 wt% CS solution. The mixture was stirred in a vortex mixer, and PIC gels were formed. The resulting PIC gels were centrifuged (6000 rpm, 5 min) and washed with ultrapure water. The supernatant and cleaning liquid were used to calculate the loading ratio of TBZ in the gels. CS/CHI/TBZ films were prepared in a manner similar to that of CS/CD–CHI films.

### 2.7. Characterization

NMR spectra were recorded on an AVANCE NEO 400 spectrometer (Bruker Biospin Corp.). The ^1^H NMR (400 MHz) spectrum was measured using 4,4-dimethyl-4-silapentane-1-sulphonic acid sodium salt (DSS) as an internal standard. DMSO-d_6_, a 2 wt% deuterium chloride in D_2_O, and a 2 wt% acetic acid-d_4_ D_2_O solution were used as NMR solvents for CDma, CD–CHI, and CD–CHI/TBZ, respectively. Fourier-transform infrared spectroscopy (FT-IR) measurements were conducted (Nicolet 380; Thermo Fisher Scientific Inc., and FT/IR 6X, JASCO Corp.). The spectra were obtained using the single reflection attenuation total-reflection (ATR) method with a Quest ATR accessory equipped with a diamond prism (GS-10800, Specac Ltd). The X-ray diffraction (XRD) patterns of the samples were obtained using an ULTIMA IV (Rigaku Corp.) with a Cu Kα source and high-speed detector under the following conditions: tube voltage of 40 kV, tube current of 40 mA, and step width of 0.02°. The XRD patterns were obtained after subtracting the background. The morphology and elemental composition of the films were evaluated using scanning electron microscopy (SEM, GeminiSEM 360; Carl Zeiss AG) at an acceleration voltage of 1 kV. In some cases, the specimens were coated with osmium using an osmium coater (Neoc-Pro; Meiwafosis Co., Ltd) to prevent charge-up. DFT calculations were performed at the B3LYP/6-31G(d) level.^52–54^ The software programs used were the Gaussian 09W (revision D.01) for the structural optimization of a model of CD–CHI. To reduce the calculation cost, the CD–CHI model consisting of five glucosamine units and a CD unit was used. The initial structure of the CD–CHI model was prepared using GaussView 5.0.9.

The mechanical properties of the films were quantitatively evaluated to calculate the tensile strength using a universal tester (Autograph AGS-500NJ; Shimadzu Corp.). First, the film thickness required to calculate the tensile strength (MPa) was measured using a micrometer (MDE-25MJ; Mitutoyo Corp.). Afterward, the films were cut into strips (1 cm × 3 cm) and placed in the apparatus while maintaining an initial gauge length of 2 cm. The stretching speed was set at 1 mm min^−1^. The obtained stress–strain curves were analyzed using the Trapezium X software (Shimadzu Corp.). The maximum tensile strengths (MPa), strain, and Young's modulus were expressed as the mean ± S.D.

### 2.8. Swelling behaviour of polysaccharide composite films

The swelling behaviour of the films was evaluated according to the methods described in previous studies.^33–35^ The films (1 cm × 1 cm) were immersed in distilled water or phosphate buffer saline (PBS, pH 7.4) and incubated at room temperature. The degree of swelling (%) and weight loss (%) were calculated using eqn (1) and (2), respectively.

1 Degree of swelling (%) = (*W*_S_ − *W*_I_)/*W*_I_ × 100

2 Weight loss (%) = (*W*_I_ − *W*_F_)/*W*_I_ × 100

*W*_I_ is the initial weight of the film before immersion, *W*_S_ is the weight of the swollen film after immersion, and *W*_F_ is the final weight of the film after the immersion experiments, followed by drying in air. *W*_S_ was obtained after every 10 min of immersion.

### 2.9. Estimation of the loading amount of TBZ in polysaccharide composite films

The loading amount of TBZ (*n*_M_) was calculated from the difference of initial TBZ (*n*_0_) from TBZ amount in supernatant (*n*_1_) and TBZ amount in washing liquid (*n*_2_) (eqn (3)). These TBZ amounts were estimated by UV-vis spectra (V-660, JASCO Corp.) using each calibration curve (Fig. S2A and D). Further, the loading ratio of TBZ was calculated by eqn (4).

3 *n*_M_ = *n*_0_ − *n*_1_ − *n*_2_

4
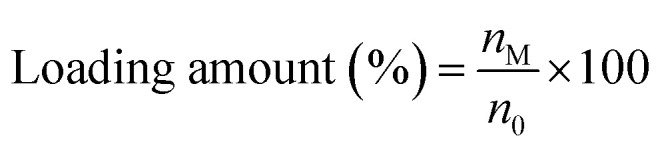


### 2.10. Release behaviour of TBZ from polysaccharide composite films

The release behaviour of TBZ from polysaccharide composite films was investigated using UV-vis spectroscopy. CS/CD–CHI/TBZ films or CS/CHI/TBZ films (1 cm × 1 cm) were immersed in 50 mL phosphate buffer saline (PBS, pH 7.4) or mixed solvent of PBS (pH 7.4) and ethanol (7 : 3 (v/v), PBS : EtOH). Then, the samples were incubated at 36.5 °C using a water bath and a stirrer. At predetermined time intervals, 1 mL of the release medium was withdrawn. The UV absorption of the released TBZ (298 nm) was measured using UV-vis spectroscopy (V-660, JASCO Corp.). The amount of released TBZ (*n*_A_) was determined using a calibration curve of TBZ in the buffer solution (Fig. S2B, C, E and F). The release ratio of TBZ was obtained using eqn (5).5
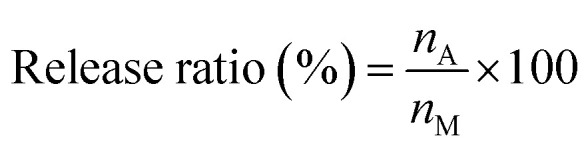


The release profile of TBZ from each film was fitted using the Higuchi and Korsmeyer–Peppas kinetics models.^55–59^ The amount of drug released after *t* hours, *Q*_*t*_, was approximated as shown in eqn (6) and (7), which corresponds to the Higuchi model and Korsmeyer–Peppas kinetics model, respectively.

6 *Q*_*t*_ = *K*_H_*t*^1/2^

7 *Q*_*t*_ = *K*_KP_*t*^*n*^

*K*_H_ and *K*_KP_ represent the release rate constants for each model. In the Korsmeyer–Peppas kinetics model, values of “*n*” around 0.5 indicate a process controlled by diffusion (Fickian diffusion).^59^ Furthermore, the determination coefficient *R*^2^ and correlation coefficient *r*^2^ were also calculated.

The monolithic solution model was also employed for fitting the release profile of TBZ from each film.^60^ The amount of drug released after *t* hours, *M*_*t*_, was approximated as shown in eqn (8) and (9) in the early and late release stages, respectively.8
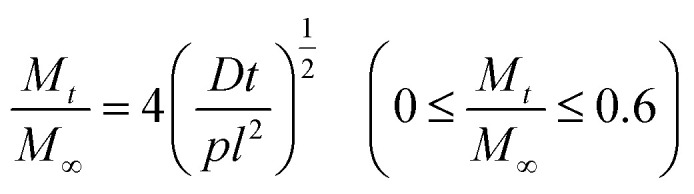
9
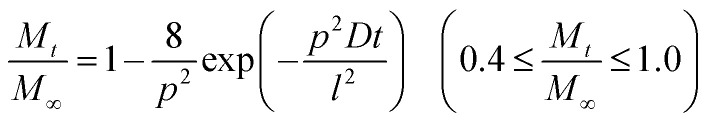
*M*_∞_ means the amount of release when it becomes constant, *D* stands for the diffusion coefficient, and *l* is the membrane thickness. The release amount is proportional to the square root of the time elapsed until 60% of the polymer matrix is released and then increases exponentially. The correlation between the calculated and experimental data was evaluated using the determination coefficient *R*^2^.^61^

A kinetic analysis of the formation and dissociation reactions of the inclusion complex between β-CD and TBZ (CD/TBZ) was also performed. In this study, we focused on the inclusion complex of the CD unit of CD–CHI and TBZ in CS/CD–CHI films. Therefore, we used the data from the release experiments as described above. The rate constant for the dissociation of CD/TBZ to form CD and TBZ is *k*_1_, and that for the formation of CD/TBZ from CD and TBZ is *k*_2_, respectively, and these reactions can be expressed as shown in eqn (10) and (11), respectively.^61^10
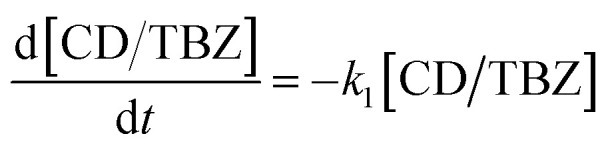
11
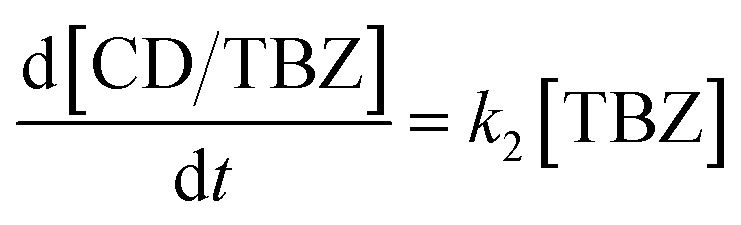
[TBZ] stands for the amount of released TBZ to PBS. Furthermore, eqn (10) was assumed to be a pseudo-first-order reaction. From eqn (10) and (11), the production rate of [CD/TBZ] is as follows.12
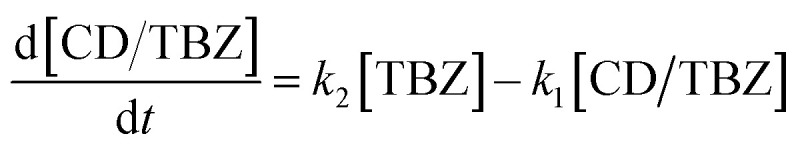


The initial concentration of CD/TBZ, [CD/TBZ]_0_, is the amount of TBZ included in the CD unit in the films; therefore, it can be expressed as [TBZ] = [CD/TBZ]_0_ − [CD/TBZ]. Substituting this equation into eqn (12), the following equation can be obtained.13
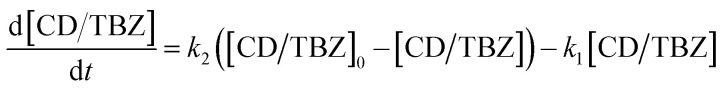
The fitting was performed using this equation with a time interval of d*t* = 0.1.

## 3. Results and discussion

### 3.1. Synthesis and characterization of CDma and CD–CHI

Initially, the synthesis of CDma from β-CD by oxidation of the 6-position of the hydroxy group with Dess–Martin periodinane (DMP) was carried out (Fig. 1B).^50,51^ The yield of CDma was 511.8 mg, and the obtained CDma was characterized by ^1^H NMR measurements. A peak at 9.52 ppm originating from H^6′^ and 4.20 ppm originating from H^5′^ in the oxidized glucose unit (Fig. S3A). Further, a peak at 4.94 ppm originating from H^1′^ appeared, and it was shifted from a peak at 4.82 ppm originating from H^1^ in β-CD (Fig. S3B). The reaction ratio of β-CD was calculated by the integration ratio between H^1^ and H^1′^.^50^ When 1.5 eq. of DMP was used as an oxidant for the oxidation of β-CD, the reaction ratio was 125%. It indicated that β-CD underwent excessive oxidation, which is different from that reported previously.^51^ It is believed that the excess oxidation occurred by the adsorbed water in β-CD or DMP, and the oxidation by Dess–Martin periodinane is known to proceed by the addition of water.^62^ The oxidative product was a mixture of CDma and β-CD derivatives with two or more formyl groups. The obtained mixture was used without further purification because CHI can react not only with CDma but also with β-CD derivatives containing two or more formyl groups.

**Fig. 3:**
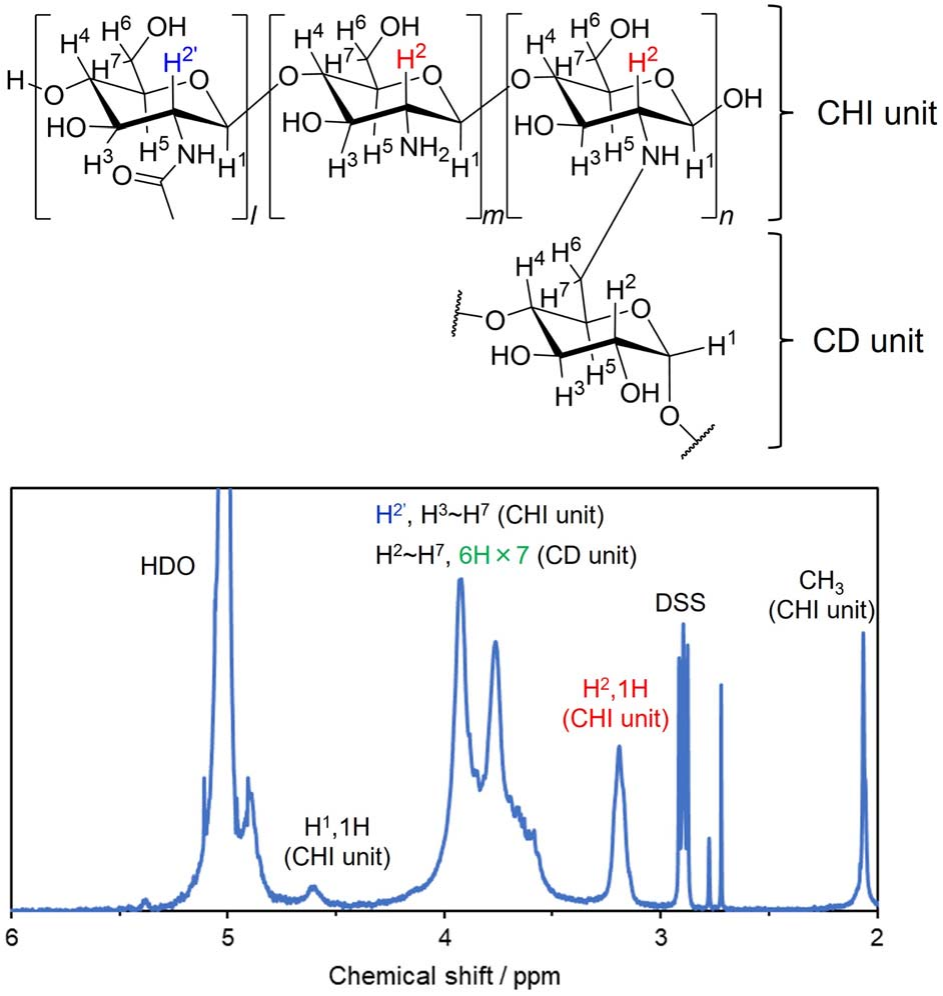
Enlarged view of the ^1^H NMR spectrum of CD–CHI. The range was expanded between 2 and 6 ppm. The modification ratio of β-CD was calculated using eqn (S1).

CD–CHI was synthesized by nucleophilic addition of an amino group in CHI to the formyl group in CDma and subsequent reductive amination (Fig. 1B and S4).^50^ The mixture of CDma was reacted with CHI in the presence of 2-picoline borane instead of sodium cyanoborohydride to give CD–CHI. The obtained CD–CHI was characterized by NMR measurements, and the modification ratio of the CD unit in CD–CHI was calculated from the integration ratio of the ^1^H NMR spectra. A signal at 3.2 ppm was characterized as H^2′^ in CHI (excluding *N*-acetylglucosamine units) and signals around 3.5–4.2 ppm were characterized as H^2″^ (*N*-acetylglucosamine units), H^3^–H^6^ of CHI and H^2^–H^6^ of CD units (Fig. 3). The intensity of the signal for one of the protons at the CD unit and the modification ratio of CD were calculated by eqn (S1). In the case of the reaction time of 1 hour, the CD modification ratio for CD–CHI was 4.3%. To improve the modification ratio, the reaction time was extended to 3, 6, and 24 h. The modification ratio increased with reaction time (3 h: 5.1%, 6 h: 5.9%, and 24 h: 9.2%), indicating that the formation of the imine between CDma and CHI needs a long reaction time. Moreover, an increase in the amount of CDma to the same amount as that of the CHI unit results in a 17.6% modification ratio of CD-CHI. This modification ratio was almost the same as that obtained from the CD–CHI synthesis *via* mono-6-O-(p-toluenesulfonyl)-β-cyclodextrin.^63^ It indicated that the nucleophilic attack of an amino group around the CD-modified glucosamine unit to a formyl group in CDma was suppressed because of steric hindrance between their CD units. To confirm such a steric effect, structural optimization of a model of the CD–CHI molecule by DFT calculation at the B3LYP/6-31G(d) level was performed. In the optimized structures, the molecular length of the four glucosamine units (18.2 Å) and the diameter of the β-CD (16.5 Å) were comparable (Fig. S5). Therefore, β-CD can be modified for every five glucosamine units of CHI, and the maximum β-CD modification ratio might be less than 20%. According to the DFT calculation, the CD modification ratio (17.6%) was a reasonable result. To further increase the modification ratio, a modification of spacers for the reduction of the steric hindrance between β-CD units should be required. It was considered that the results of the CD modification ratio were sufficient to prepare CD-containing polysaccharide composite films.

### 3.2. Preparation, characterization and evaluation of CS/CD–CHI films

**Fig. 4:**
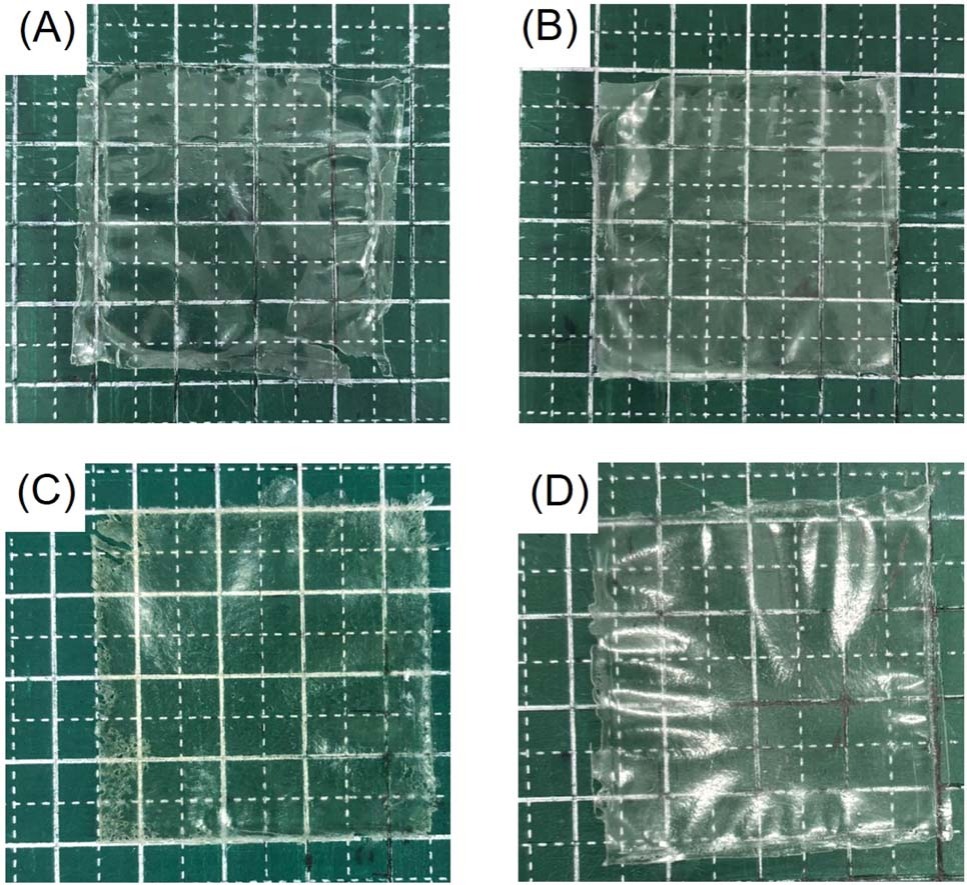
Photographs of (A) CS/CD–CHI film, (B) CS/CHI film, (C) CS/CD–CHI/TBZ film, and (D) CS/CHI/TBZ film (grid: 1 × 1 cm). These films were hot-pressed at 35 °C.

CS/CD–CHI films were fabricated from polyion complex gels (PICs) consisting of CS and CD–CHI using hot-press techniques. Regarding CD–CHI, the CD modification ratio of 9.2% was used. Mixing the CS and CD–CHI solutions yielded PICs, and it was similar to the formation of PICs comprising CS and CHI.^27,28^ When the obtained PICs were hot-pressed at 120 °C, deep yellow films with cracks were obtained, indicating that the Maillard reaction had occurred in the films (Fig. S6).^64^ To prevent the Maillard reaction, the hot pressing was conducted at 35 °C, and transparent and free-standing films were obtained (Fig. 4A and B). The yield of the CS/CD–CHI film was 0.159 ± 0.003 g (82.9% ± 1.7%), which was similar to the CS/CHI films (0.166 ± 0.002 g, 85.1% ± 0.7%). Here, an excess amount of CS was used for the formation of PICs; therefore, the used CD–CHI should be incorporated into the obtained gels. Based on this assumption, the amount of CD units in the films was approximately 25.6 µmol. Further, the thicknesses of the CS/CD–CHI films and the CS/CHI films were 69 ± 3 µm and 73 ± 3 µm, respectively, suggesting that the thicknesses were nearly the same whether the CD was included or not in the films. These thicknesses differed from that of a PET sheet spacer (100 µm). Previously, CS/CHI films prepared by hot pressing at 35 °C showed a reduction in their thickness.^34^ When the CS/CHI film was prepared at 35 °C, a certain amount of water remained in the PIC gel, and the swollen films having the thickness of the spacer were obtained. Subsequently, the films were dried, losing moisture and shrinking to a lower thickness. As a control experiment, CS/CHI films, including β-CD and CD/CS/CHI films, were prepared. CS solutions were added to mixed solutions of β-CD and CHI to form PIC gels, and the amount of incorporated β-CD was evaluated by HPLC measurement of the supernatant and washing solution. The amount of β-CD in CD/CS/CHI films should be almost the same as that of β-CD in the PIC gels because of the lack of a washing process in the film formation procedure. Therefore, the amount of β-CD in CD/CS/CHI films was determined from the supernatant and washing solution obtained during the PIC gel preparation procedure using HPLC analysis (Fig. S1). The loaded ratio of β-CD in the films was 0.5%, indicating that most of the β-CD was not incorporated, suggesting that the preparation of CD/CS/CHI films is difficult.

The characterization and surface morphology of the obtained CS/CD–CHI films were evaluated using FT-IR spectroscopy and SEM. FT-IR spectra of CS/CD–CHI films, CS/CHI films, CS, CHI, and β-CD are shown in Fig. S7. The CS/CD–CHI films and CS/CHI films showed a peak around 1530 cm^−1^ originating from NH_3_^+^ groups, which confirmed the formation of PICs.^28^ The spectra of CS/CD–CHI films and CS/CHI films were almost the same, and the peaks derived from β-CD were not confirmed. This is because the peaks of β-CD, CS, and CHI were overlapped, and the modification ratio of β-CD in CD–CHI was low (9.2%). The surface morphologies of CS/CD–CHI films and CS/CHI films were evaluated by SEM observation (Fig. S8). The SEM images showed that both films had dense, smooth surfaces, and it is suggested that the presence or absence of CD did not affect the surface morphology.

**Fig. 5:**
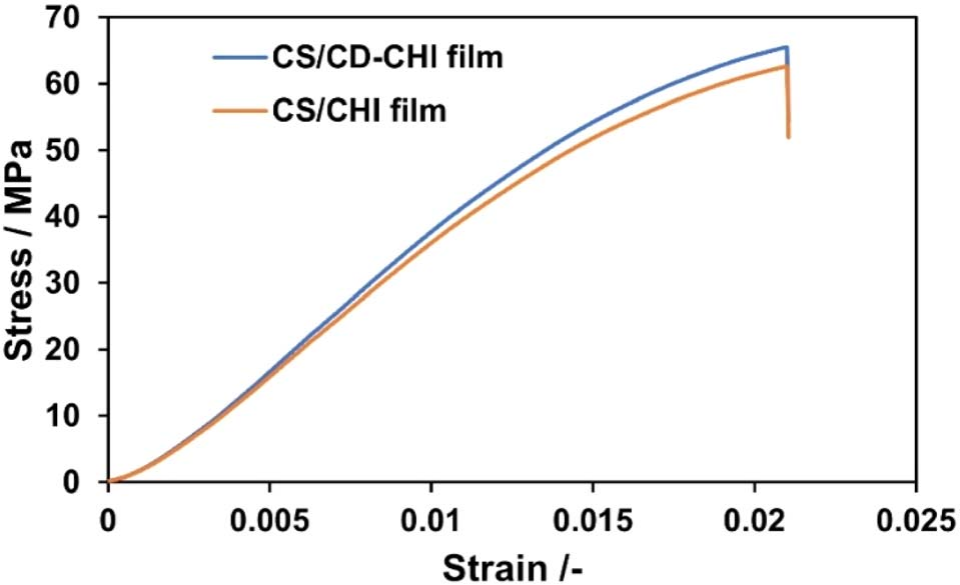
Stress–strain curves of CS/CD–CHI films and CS/CHI films.

**Fig. 6:**
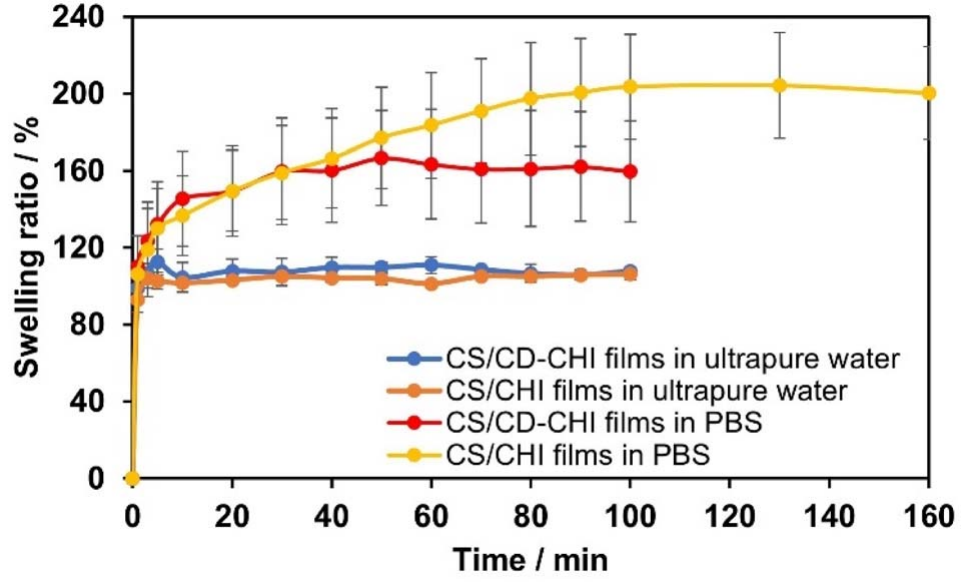
Swelling behaviour of the CS/CD–CHI films and CS/CHI films in ultrapure water or PBS.

The mechanical and swelling properties of CS/CD–CHI films were also evaluated. Initially, tensile tests and film thickness measurements were carried out. The stress–strain curves of the CS/CD–CHI and CS/CHI films were comparable (Fig. 5). The maximum stresses, the ultimate elongation, and the Young's modulus of CS/CD–CHI and CS/CHI films were 75.2 ± 16.2 MPa, 3.0% ± 0.6%, and 1866 ± 25 MPa, and 66.8 ± 3.0 MPa, 4.1% ± 0.3%, and 1714 ± 413 MPa, respectively. These results suggested that the use of CD–CHI did not affect the mechanical properties of the films. Moreover, the swelling behaviour of the films in ultrapure water and phosphate-buffered saline (PBS, pH 7.4) was also investigated (Fig. 6). The swelling ratios of the CS/CD–CHI films and CS/CHI films after 100 min immersion in ultrapure water were almost the same, at 109.4% ± 11.9% and 106.1% ± 3.0%, respectively. On the other hand, the swelling ratios of the CS/CD–CHI and CS/CHI films after 100 min immersion in PBS were 159.7% ± 26.2% and 200.2% ± 24.2%, respectively. PBS contains inorganic salts, and therefore, electrostatic interactions between polysaccharides were weakened when the film was immersed in PBS, which could allow the films to incorporate more water.^29^ Further, the swelling ratios of the CS/CD–CHI and CS/CHI films did not show significant differences (*p > 0.05 was obtained by the *t*-test in both media). Accordingly, the modification of CD units did not affect the mechanical and swelling properties.

### 3.3. Fabrication of CS/CD–CHI/TBZ films

**Fig. 7:**
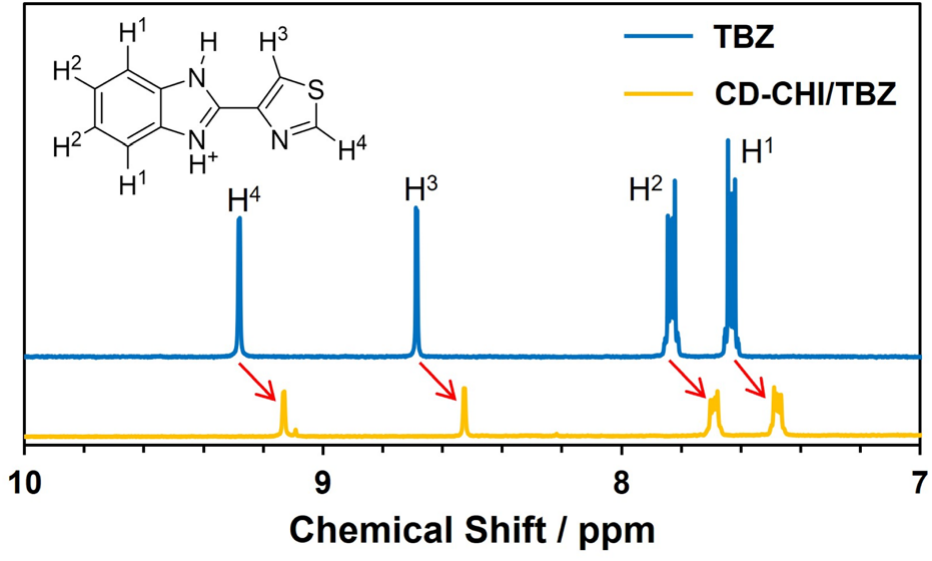
^1^H NMR spectra of TBZ and CD–CHI/TBZ in the 2% acetic acid-d_4_ D_2_O solution.

**Fig. 8:**
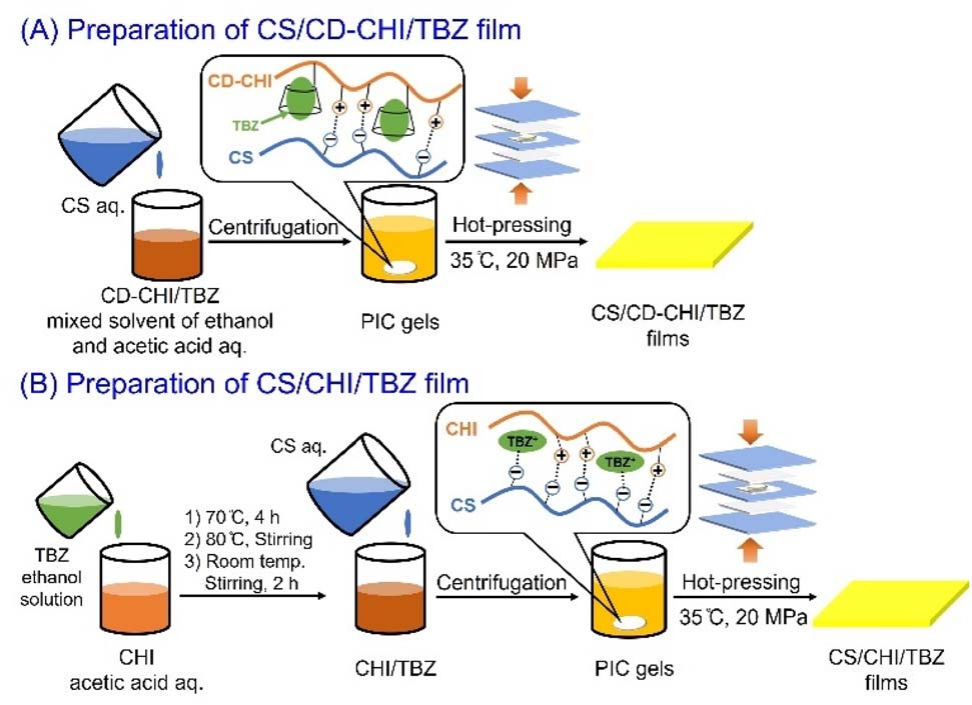
Schematic illustration of the preparation of (A) CS/CD–CHI/TBZ film and (B) CS/CHI/TBZ film.

For the evaluation of the molecular loading ability of the CD unit in CD–CHI, the preparation of thiabendazole (TBZ)-loaded CS/CD–CHI films, denoted as CS/CD–CHI/TBZ films, was performed (Fig. S10). Here, TBZ was used as a hydrophobic drug molecule that can form inclusion complexes with β-CD.^42^ CS/CD–CHI/TBZ films were prepared using TBZ-encapsulated CD–CHI, CD–CHI/TBZ. The inclusion of TBZ in the CD unit of CD–CHI/TBZ was confirmed by a shift in the signals originating from TBZ in the ^1^H NMR spectra (Fig. 7) similar to an inclusion complex between β-CD and TBZ.^42^ CS/CD–CHI/TBZ films were obtained by hot press techniques from the PICs formed by mixing CD–CHI/TBZ solutions with CS solutions (Fig. 8A). Further, TBZ-loaded CS/CHI films, denoted as CS/CHI/TBZ films, were also prepared as a control experiment (Fig. 8B). TBZ was poorly soluble in water and acetic acid solution, while it was soluble in a mixed solvent of ethanol and low pH aqueous solution. Furthermore, the loaded TBZ should be protonated to become a monocation (p*K*_a_ 4.2, TBZ^+^) or a dication (p*K*_a_ 2.5, TBZ^++^),^65,66^ which can interact with anionic groups in CS by electrostatic interactions (Fig. S9). Macroscopic observation of the prepared CS/CD–CHI/TBZ and CS/CHI/TBZ films revealed that both were transparent and free-standing (Fig. 4C and D). The amounts of loaded TBZ in the CS/CD–CHI/TBZ films and CS/CHI/TBZ films were 22.0 ± 4.7 µmol (60.1% ± 12.8%) and 24.5 ± 6.2 µmol (68.9% ± 17.4%), respectively. Even for the case of CS/CHI/TBZ films, changing the loading solution condition resulted in a significant improvement of the loading amount, compared to the previous report, which showed that the loading amount of TBZ was less than 50 nmol when an aqueous solution was used as the solvent.^34^ On the other hand, an excess amount of CS was also used when the CS/CD–CHI/TBZ films were fabricated, and the amount of CD units in the CS/CD–CHI/TBZ films was approximately 25.6 µmol. Therefore, approximately 85.9% of the CDs in the CS/CD–CHI/TBZ film should be utilized as inclusion complexes with the loaded TBZ. Moreover, in the absorption spectra of the films, a peak originating from TBZ was observed at 300 nm in both films (Fig. S10). Although almost the same amount of TBZ was loaded, the UV-vis absorption spectrum of the CS/CD–CHI/TBZ film had a higher spectral intensity at 300 nm than that of the CS/CHI/TBZ films. It indicated that inclusion complexes between the CD unit of CD–CHI and TBZ were formed in the CS/CD–CHI/TBZ films.^42^

To further evaluate the formation of the inclusion complexes between TBZ and β-CD in the CS/CD–CHI/TBZ film, SEM observation and powder XRD measurement were performed. SEM images of the CS/CD–CHI/TBZ films showed that deposits of about 30 µm were found on their surfaces, suggesting that part of the TBZ molecules that were not captured by the CD units aggregated and formed crystals in the films (Fig. S13A). Some of them might also be deposited and crystallized during the preparation of SEM samples under dry conditions. These results suggested that CS/CD–CHI/TBZ had not only encapsulated TBZ but also a certain amount of decapsulated TBZ. On the other hand, CS/CHI/TBZ films also showed deposits, which might be TBZ crystals on the film surface (Fig. S13B). Furthermore, powder XRD measurement of both CS/CD–CHI/TBZ films and physical mixtures of CS/CHI–CD and TBZ revealed the pattern originating from TBZ crystals (Fig. S14), which also supported that the observed deposits on/in the films were TBZ crystals. From the above, the observed precipitates in the SEM images might be TBZ crystals, and the XRD results also supported this.

### 3.4. Evaluation of the release behaviour of TBZ and elucidation of its mechanism

**Fig. 9:**
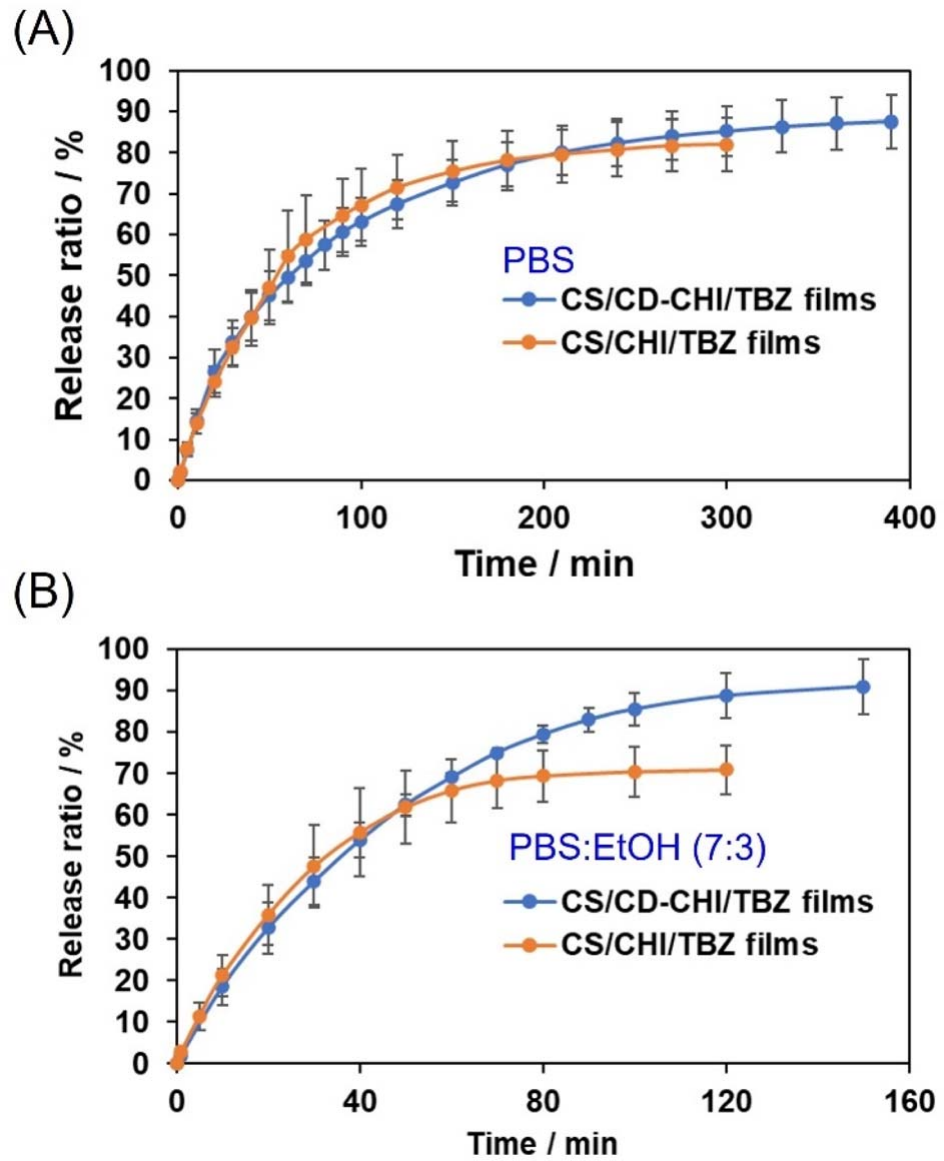
Release behaviour of TBZ from the CS/CD–CHI/TBZ films or CS/CHI/TBZ films in (A) PBS and (B) PBS : EtOH (7 : 3 (v/v)).

The release behaviour of TBZ from the CS/CD–CHI/TBZ films and the CS/CHI/TBZ films in PBS (pH 7.4) and a mixed solvent of PBS (pH 7.4) and ethanol (PBS : EtOH (7 : 3 (v/v))) was examined (Fig. 9). The release ratio of TBZ in the CS/CD–CHI/TBZ films gradually increased and reached 87.4% ± 6.5% at 400 minutes, and most of the loaded TBZ was released. On the other hand, the release ratio of TBZ in the CS/CHI/TBZ films was 82.0% ± 6.5% at 300 minutes, which was faster to reach an equilibrium than that of the CS/CD–CHI/TBZ films. It is assumed that the release of TBZ from the CS/CD–CHI/TBZ films proceeded *via* two steps: dissociation of inclusion complexes between TBZ and the CD unit, followed by dispersion in the films, resulting in slow release. To investigate the effects of the inclusion complexes between TBZ and the CD unit on the release behaviour of TBZ, the release of TBZ from these films in PBS : EtOH (7 : 3 (v/v)) was also examined. The use of PBS : EtOH as a medium should promote dissociation of the inclusion complex compared to PBS alone.^42^ The release ratio of TBZ in the CS/CD–CHI/TBZ film was 92.5% ± 7.3% at 150 minutes and then remained constant, indicating that the release of TBZ in PBS : EtOH was faster than that in PBS. When the CS/CHI/TBZ film was used, the release ratio was 70.9% ± 5.8% at 120 minutes, which was also faster to reach an equilibrium than that of the CS/CD–CHI/TBZ films. These results indicate that TBZ within each film was easily dissolved by ethanol in the medium.

The release mechanism of TBZ from CS/CD–CHI/TBZ films was investigated using two mathematical models: Higuchi and Korsmeyer–Peppas kinetics models (Table 1 and Fig. S15 and S16). The Higuchi model (eqn (6)) is used to explain the release of drugs from homogeneous systems, following a Fickian diffusion mechanism.^55,56^ The model was applied to both films and media, and the *R*^2^ and *r*^2^ values showed that, in each case, they were generally consistent with the Higuchi model (Table 1). The Korsmeyer–Peppas model (eqn (7)) was also used to estimate the drug release mechanism, and all cases followed this model (Table 1). Additionally, the *n* value is known to be important for understanding the release mechanism. In the case of CS/CD–CHI/TBZ in PBS, CS/CHI/TBZ in PBS, and CS/CD–CHI/TBZ in PBS : EtOH, the *n* values were lower than 0.5 (Table 1), indicating that these films were consistent with quasi-Fickian diffusion.^58,59^ Meanwhile, CS/CHI/TBZ in PBS : EtOH showed *n* = 0.5210 (Table 1), which approximated the Higuchi model (*n* = 0.5). It suggested that this condition roughly followed Fickian diffusion. Accordingly, the release behaviour of the CS/CD–CHI/TBZ film was different from that of the CS/CHI/TBZ film.

**Table 1:** Kinetic analysis of the TBZ release from polysaccharide composite films

Conditions	Higuchi			Korsmeyer–Peppas			
	*K*_H_	*R* ^2^	*r* ^2^	*K*_KP_	*n*	*R* ^2^	*r* ^2^
CS/CD–CHI/TBZ (PBS)	4.7502	0.9684	0.9378	14.4236	0.3312	0.9873	0.9704
CS/CHI/TBZ (PBS)	5.3369	0.9637	0.9288	13.4934	0.3509	0.9903	0.9543
CS/CD–CHI/TBZ (PBS : EtOH)	7.3872	0.9631	0.9276	15.7901	0.3674	0.9743	0.9493
CS/CHI/TBZ (PBS : EtOH)	7.7575	0.9774	0.9553	6.9878	0.5210	0.9776	0.9557

Moreover, the release profiles were fitted to the monolithic solution model, which is also used to model drug release from slab-type reservoirs, such as thin films.^60^ The release behaviour of TBZ in PBS was plotted using eqn (8) and (9). Eqn (8) holds as an early-time fitting model, remaining accurate for up to 60% of the total release, 0 ≤ *M*_*t*_/*M*_∞_ ≤ 0.6, and eqn (9) holds as the late-time fitting for the final stages of release, 0.4 ≤ *M*_*t*_/*M*_∞_ ≤ 1.0. These fitting results are shown in Fig. S17. The CS/CD–CHI/TBZ film and CS/CHI/TBZ showed that the early- and late-time fittings were correlated with the experimental data in both media (*R*^2^ > 0.99 in each case). Notably, it was known that the fitting to this model affords an apparent diffusion coefficient *D*.^60^ The *D* value of CS/CD–CHI/TBZ in PBS (*D* = 2.2 × 10^−11^) was lower than that of CS/CHI/TBZ in PBS (*D* = 3.1 × 10^−11^). Further, the tendency of the *D* value also applied to the TBZ release in PBS : EtOH (CS/CD–CHI/TBZ: *D* = 4.2 × 10^−11^, CS/CHI/TBZ: *D* = 6.6 × 10^−11^, respectively). These results indicated that the fitting to the monolithic solution model showed different release behaviour between the CS/CD–CHI/TBZ and CS/CHI/TBZ films.

The difference in release behaviour between with and without β-CD units should be due to the formation of inclusion complexes of β-CD with TBZ. To confirm the TBZ release from the CD units in the CS/CD–CHI/TBZ film, the pseudo-first-order rate constant of dissociation (*k*_1_) and formation (*k*_2_) of the CD/TBZ inclusion complex by curve-fitting of the actual experimental data was performed (eqn (13)). When TBZ was released in PBS, the kinetic constants were *k*_1_ = 0.012 h^−1^ and *k*_2_ = 0.0018 h^−1^ (Fig. S18A). On the other hand, a curve-fitting for the release behaviour of CS/CD–CHI/TBZ in PBS : EtOH yielded the kinetic constants *k*_1_ = 0.020 and *k*_2_ = 0.00095 (Fig. S18B). It suggested that the dissociation rate of CD/TBZ in PBS was faster than that in PBS : EtOH, and the formation rate of the inclusion complex in PBS was slower than that in PBS : EtOH. From the above, the release of TBZ follows a pseudo-first-order reaction of the CD unit and TBZ. From these kinetic studies, TBZ was released into the solution without being retained in the film after its release from the CD units. Moreover, it was also suggested that the release of TBZ from the CD unit was the rate-limiting step in the release mechanism, and the release behaviour of TBZ depended on the decomposition rate of CD/TBZ. These mechanisms differ from the drug release mechanism of drug-loaded CS/CHI films (including CS/CHI/TBZ films), which are released from polysaccharide chains.^29,34^

Based on the above results of film fabrication, TBZ release behaviour, and kinetic analysis, we propose a release mechanism of TBZ from each film. In the case of CS/CD–CHI/TBZ films, most of the TBZ molecules are incorporated within the CD units. Therefore, they are uniformly dispersed within the films. During TBZ release, dissociation of the inclusion complex occurs, followed by sustained release through the formation and dissociation of the inclusion complex with CD in the carrier. The release occurs regardless of the solvent type, resulting in the same release behaviour in both PBS and PBS–EtOH. On the other hand, in the case of CS/CHI/TBZ films, the TBZ is precipitated as crystals within the film. Furthermore, TBZ is neutral in an aqueous solution at pH 7.4 (Fig. S9), and there is no electrostatic interaction between the TBZ and polysaccharides in the films. In other words, the release mechanism of TBZ in CS/CHI/TBZ films should depend on the solubility of TBZ. When PBS is used, TBZ gradually dissolves. Meanwhile, in the case of PBS–EtOH, TBZ dissolves rapidly and is released from the film. Therefore, we believe that the release behaviour is different between PBS and PBS–EtOH when using CS/CHI/TBZ.

Finally, the release behaviour of CS/CD–CHI films was compared to CS/CHI films or similar systems. In almost all cases of using CS/CHI films, loaded drugs were released within 60 minutes due to initial burst release.^29,34^ In contrast, the CS/CD–CHI films have the advantage of a slower release rate because initial burst release does not occur. Additionally, in comparison to drug carriers without CD units, those containing CD units have the advantage of being capable of controlling the amount of drug loaded by adjusting the number of CD units.^41^ Furthermore, compared to drug carriers containing CD units, the swelling behaviour derived from polysaccharide PICs might provide the potential to control drug release.^34,41^ However, the CS/CD–CHI films have the disadvantage that they cannot be used for large molecules, which do not form inclusion complexes with cyclodextrin. Moreover, sustained release capabilities should be further studied and will be reported in the future.

## 4. Conclusions

In this study, CD–CHI with a β-CD modification ratio of 9.2% was synthesized, and the polysaccharide composite film using CD–CHI (CS/CD–CHI film) was prepared. The mechanical strength and swelling ratio in ultrapure water of the obtained CS/CD–CHI film were comparable to those of CS/CHI. Further, the loading and sustained release ability of TBZ, as a model drug, onto the CS/CD–CHI film was confirmed. Most of the TBZ in the CS/CD–CHI/TBZ film was loaded by forming an inclusion complex with β-CD. Moreover, the release behaviour of the CS/CD–CHI/TBZ film was consistent with quasi-Fickian diffusion, and the release rate was found to correlate with the dissociation rate of the CD/TBZ inclusion complex. These results suggest that the CS/CD–CHI film can be used as a hydrophobic drug carrier with tunable loading amount.

## Author contributions

Takuya Sagawa: writing – original draft, conceptualization, supervision, data curation, writing – review & editing; Aoi Kashiwabara: investigation, methodology, visualization; and Mineo Hashizume: project administration, conceptualization, supervision, writing – review & editing.

## Conflicts of interest

There are no conflicts to declare.

## Supplementary Material

RA-015-D5RA07595E-s001

## Data Availability

Raw data supporting our findings are available from the corresponding authors upon reasonable request. All data supporting the findings of this study are available within the article and its supplementary information (SI). Supplementary information (SI): experimental procedure for synthesis of CDma and CD-CHI, UV-Vis spectra and calibration curves of TBZ solutions, ^1^H NMR spectra of CD and CDma, ^1^H NMR spectra of CD and CD-CHI, optimized structure of the model of CD-CHI, photographs of each gel and film, FT-IR spectra of each substrate and film, SEM images of each film, strass-strain curves of each film, schematic illustration of the preparation of each film, ionization of TBZ, UV-vis spectra of each TBZ-loaded film, SEM images of each TBZ-loaded film, XRD patterns of each TBZ-loaded film, analyses of TBZ release profiles from each TBZ-loaded film, fitting for release behavior of TBZ by the monolithic solutions model, and Curve-fitting of the formation and dissociation reaction of CD/TBZ inclusion complex. See DOI: https://doi.org/10.1039/d5ra07595e.
