# Supplementary material for: Preparation of polysaccharide composite films using cyclodextrin-conjugated chitosan for sustained release of hydrophobic drugs
Source: RSC Adv. 2025 Nov 24;15(54):46152–63. doi: 10.1039/d5ra07595e (PMC12643134; doi:10.1039/d5ra07595e)
Supplement: RA-015-D5RA07595E-s001 [file RA-015-D5RA07595E-s001.pdf]

Supplementary Information (SI) for RSC Advances.
This journal is © The Royal Society of Chemistry 2025

Preparation of Polysaccharide Composite Films Using Cyclodextrin-Conjugated Chitosan for Sustained Release of Hydrophobic Drugs

Takuya Sagawa,[*,a,b] Aoi Kashiwabara,[a] Mineo Hashizume[*,a,b]*

[a] Department of Industrial Chemistry, Faculty of Engineering, Tokyo University of Science, 6-3-1 Niijuku, Katsushika-ku, Tokyo, 125-8585, Japan

[b] Graduate School of Engineering, Tokyo University of Science, 6-3-1 Niijuku, Katsushika-ku, Tokyo, 125-8585, Japan

KEYWORDS: free-standing film, polysaccharide, polyion complex, β-cyclodextrin-conjugated chitosan, drug carrier

–Totals– 16 pages, 18 Figures

Table of Contents

Synthesis of β-CD monoaldehyde (CDma). ········ S3
Synthesis of β-CD-conjugated chitosan (CD-CHI). ········ S3
Fig. S1 HPLC chart and a calibration curve obtained using HPLC. ········ S5
Fig. S2 UV-Vis spectra and calibration curves of TBZ solutions. ········ S6
Fig. S3 ^{1}H NMR spectra of CD and CDma ········ S7
Fig. S4 ^{1}H NMR spectra of CD and CD-CHI ········ S7
Fig. S5 Optimized structure of the model of CD-CHI ········ S8
Fig. S6 Photographs of CS/CD-CHI gel, CS/CHI gel, CS/CD-CHI film, and CS/CHI film. ········ S8
Fig. S7 IR spectra of β-CD, CS, CHI, CS/CHI film, and CS/CD-CHI film. ········ S9
Fig. S8 SEM images of CS/CD-CHI film and CS/CHI film. ········ S10
Fig. S9 Ionization of TBZ. ········ S10
Fig. S10 UV-Vis spectra of CS/CD-CHI/TBZ film and CS/CHI/TBZ film. ········ S10
Fig. S11 SEM images of CS/CD-CHI/TBZ film and CS/CHI/TBZ film. ········ S11

Fig. S12 XRD patterns of CS/CD-CHI film and TBZ (Physical mixture), CS/CD-CHI/TBZ film, and TBZ. ········ S11
Fig. S13 Analyses of TBZ release profiles from CS/CD-CHI/TBZ films ········ in PBS. ········ S12
Fig. S14 Analyses of TBZ release profiles from CS/CHI/TBZ films in PBS. ········ S12
Fig. S15 Analyses of TBZ release profiles from CS/CD-CHI/TBZ films in PBS:EtOH. ········ S13
Fig. S16 Analyses of TBZ release profiles from CS/CHI/TBZ films in PBS:EtOH. ········ S13
Fig. S17 Fitting for release behaviour of TBZ by the monolithic solutions model ········ S14
Fig. S18 Curve-fitting of the formation and dissociation reaction of CD/TBZ inclusion complex by eq. (15). ········ S15

Synthesis of β-CD monoaldehyde (CDma).

β-CD monoaldehyde (CDma) was synthesized by previous method with several modification.[1,2] The synthesis was carried out under argon atmosphere. β-CD（565.9 mg, 0.499 mmol） and DMP (320.0 mg, 0.754 mmol) was dissolved in 13.3 mL of DMSO, and the mixture was stirred at room temperature for 2 hours. After that, the mixture was added to −20 °C of acetone to precipitate products. The mixture was centrifugated (6000 rpm, 3 min) and then filtered under reduced pressure using a membrane filter (0.2 mm, ADVANTEC, T020A047A). The centrifugation and filtration were repeated two times. The obtained solid was dissolved by ultrapure water, and the solution was filtrated using a filter (ADVANTEC, 00021125) The filtrate was lyophilized to give white solid (511.8 mg). The obtained solid was analyzed by ^{1}H NMR (Fig. S1).

^{1}H NMR (400 MHz, DMSO-d_6) δ = 9.52 (s, 1H, $H^{6'}$), 4.94 (d, J = 2.9, 1H, $H^{1'}$), 4.83 (d, J = 2.7, 6H, H^1), 4.20 (d, J = 10.5, 1H, $H^{5'}$) 3.31–3.64 (m, 5H, 33H, H^2–H^6).

Synthesis of β-CD-conjugated chitosan (CD-CHI)

CD-CHI was synthesized by previous method with several modifications.[1] CHI (151.9 mg) was dissolved in 2.0% acetic acid aqueous solution. Then, CDma (113.3 mg, 0.100 mmol) was added to the solution, and the mixture was stirred at room temperature for predetermined time (1, 3, 6, and 24 hours). After that, 2-picolineborane was added to the mixture after grinding in a mortar, and the solution was stirred at room temperature for an hour. After the reaction, NaOH solution was added to precipitate the gel-like solid. The mixture was centrifugated (6000 rpm, 5 min), filtrated under reduced pressure using a membrane filter (0.2 mm, ADVANTEC, T020A047A), and washed by ultrapure water and methanol. The obtained gel was freeze-dried, and a white solid (150.8 mg; 24 hours) was obtained. The white solid was analyzed by ^{1}H NMR spectroscopy. Before the measurement of ^{1}H NMR, the white solid was dissolved in 2% DCl/D_2O solution, and the volatility was removed under reduced pressure. Consequently, 2% DCl/D_2O solution was added, and NMR

spectrum was measured. The modification ratio of obtained CD-CHI was calculated by Eq. (S1), which was different way compared to the previous reports.[3]

$$\text{Modification ratio} = \frac{(\text{H}^{2\text{–}7}\text{-CD-CHI}) - \{(\text{H}^{2}\text{-CHI}) + 0.14\times(\text{H}^{2}\text{-CHI})\}\times 5 - 0.14\times(\text{H}^{2}\text{-CHI})}{42} \times 100 \quad \text{(S1)}$$

$H^{2–7}$-CD-CHI stands for the total integral intensity of 2-position of *N*-acetyl-D-glucosamine unit in CHI unit, 3–7-position in CHI unit, and 2–7-position in β-CD unit (3.50–4.30 ppm) (Fig. 3). H^{2}-CHI stands for the integral intensity of 2-position of D-glucosamine unit in CHI (3.12–3.24 ppm). The value of 0.14 means the deacetylation degree of using CHI. The value of 42 means the number of protons of 2–7-position in β-CD unit.

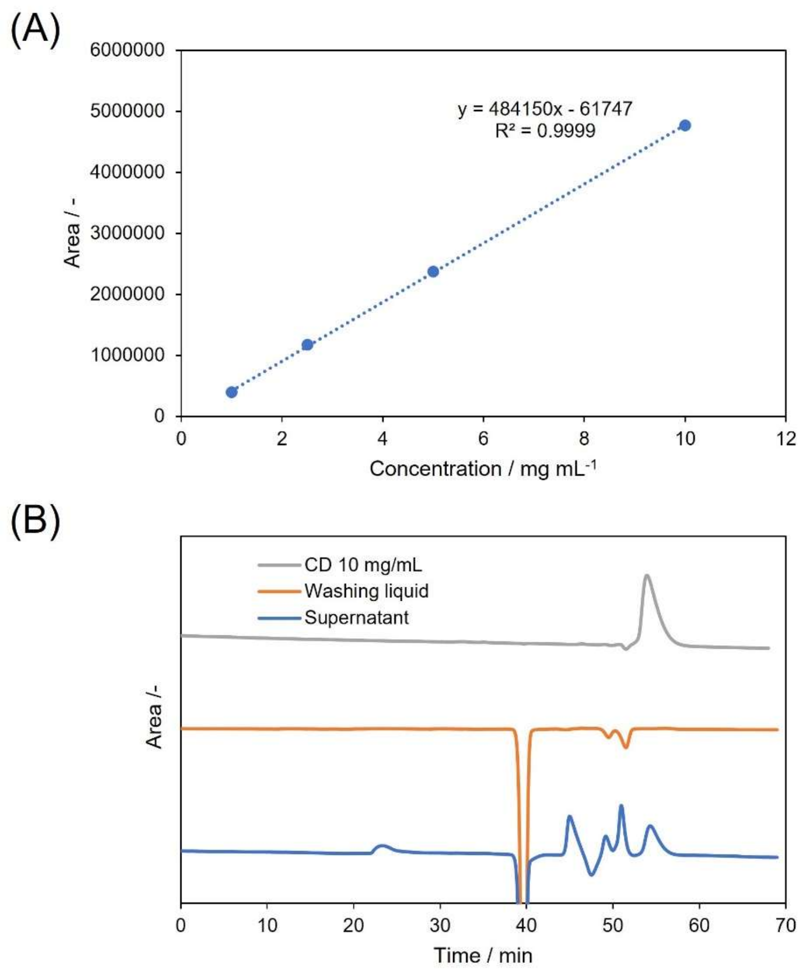

Fig. S1. (A) A calibration curve of β-CD obtained using HPLC. (B) HPLC chart of β-CD, washing liquid, and supernatant.

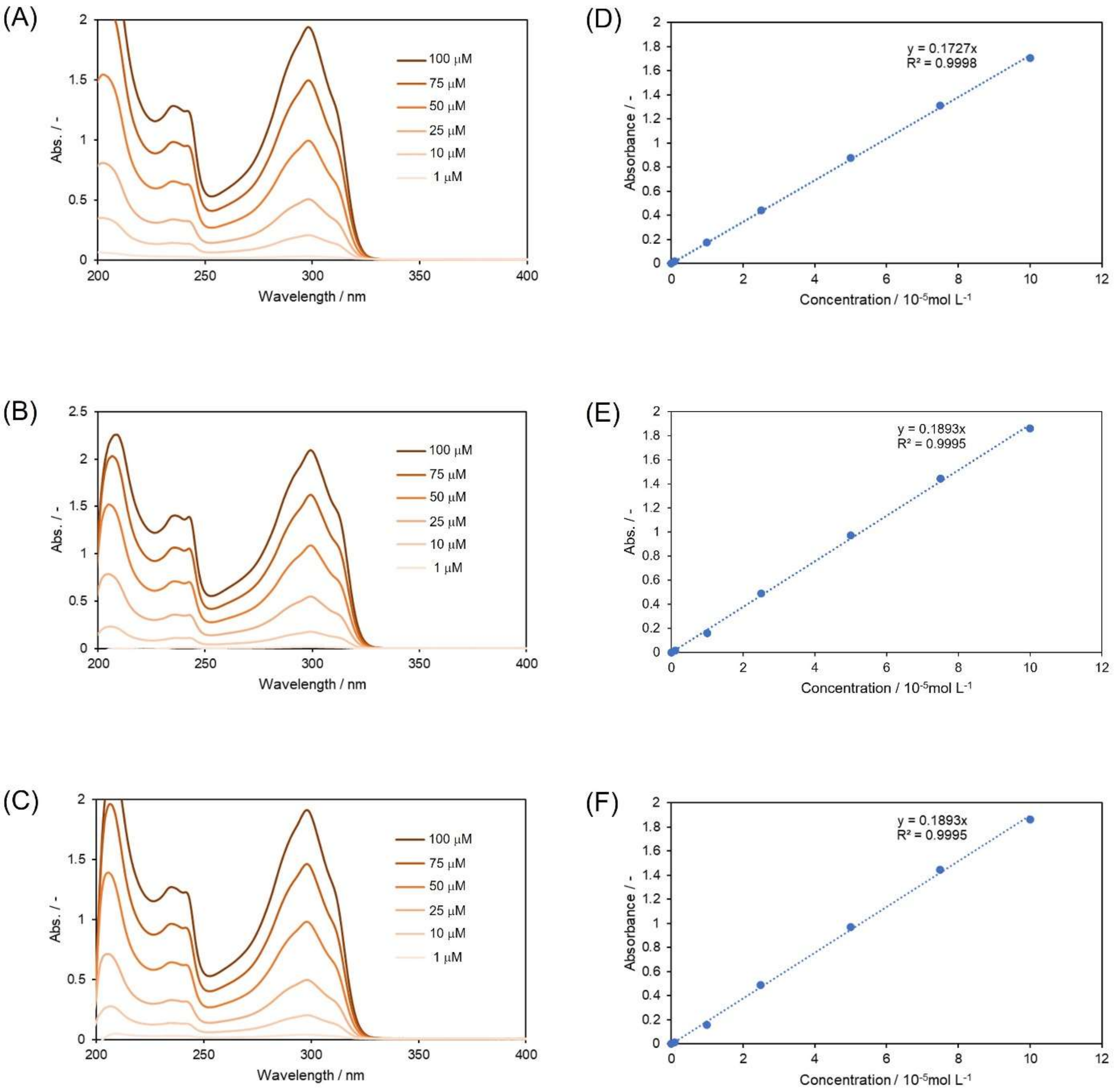

Fig. S2. UV-Vis spectra at 1, 10, 25, 50, 75, 100 mM of TBZ solution in (A) ultrapure water (B) PBS (pH 7.4), and (C) PBS:ethanol = 7:3 (v/v), and calibration curves of (D) ultrapure water (E) PBS (pH 7.4), and (F) PBS:ethanol = 7:3 (v/v).

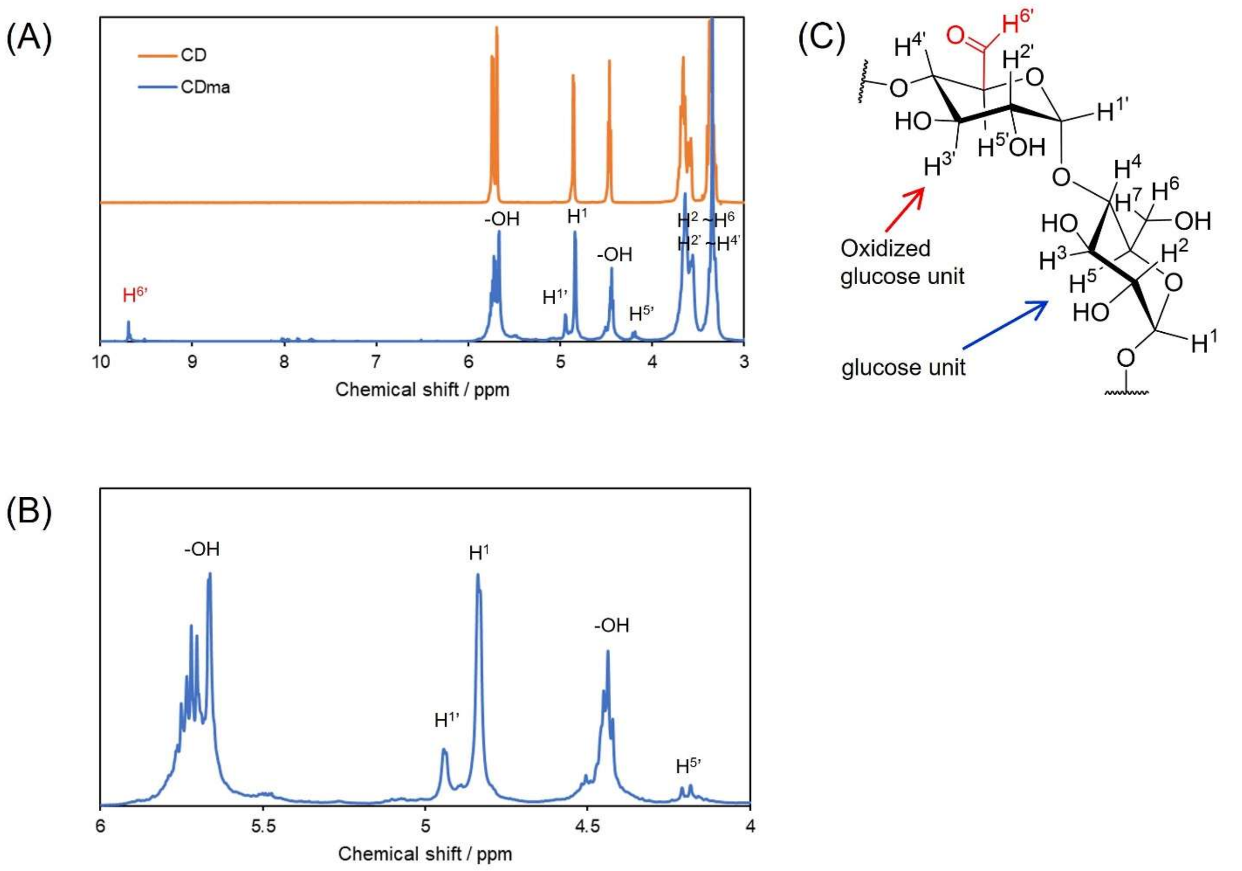

Fig. S3. (A) ^{1}H NMR spectra of CD and CDma (DMSO-d6). (B) Enlarged view of ^{1}H NMR spectrum of CDma. (C) Partial structure of CDma.

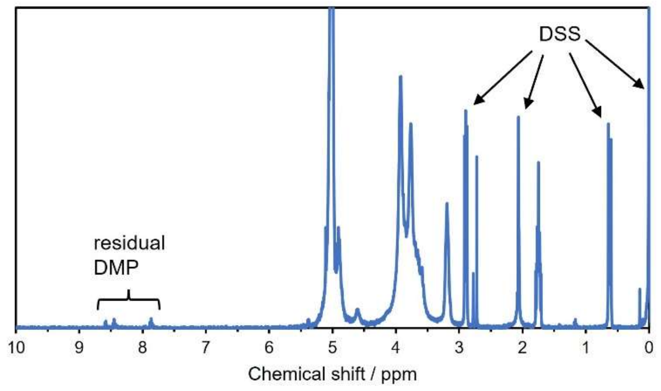

Fig. S4. ^{1}H NMR spectra of CD-CHI (2wt% deuterium chloride in D_2O).

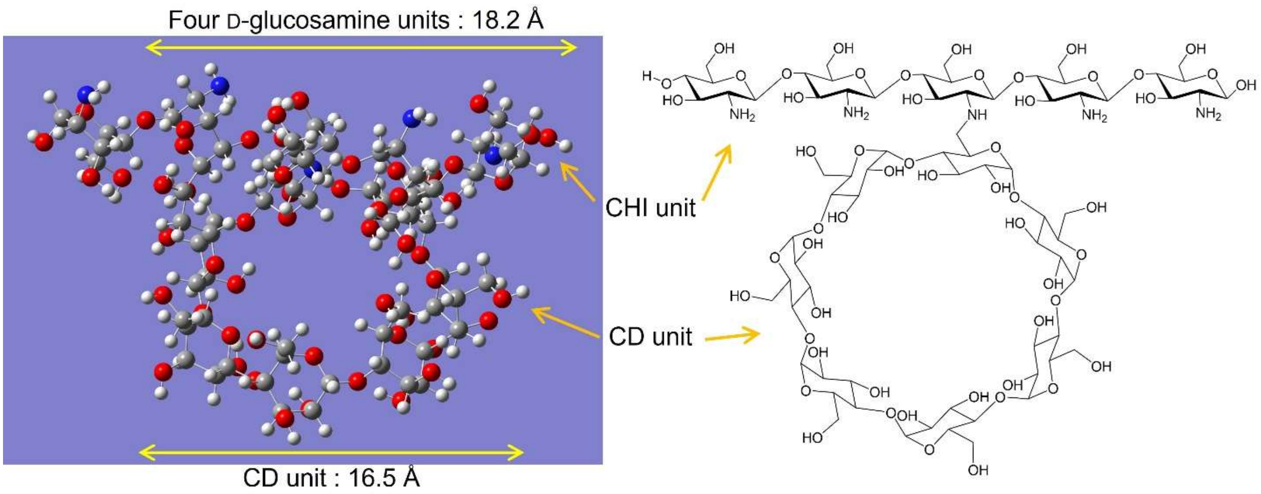

Fig. S5. Optimized structure of the model of CD-CHI composed of five D-glucosamine units and a β-CD unit (left) and its chemical structure (right). The DFT calculations were performed at the B3LYP/6-31G(d) level.

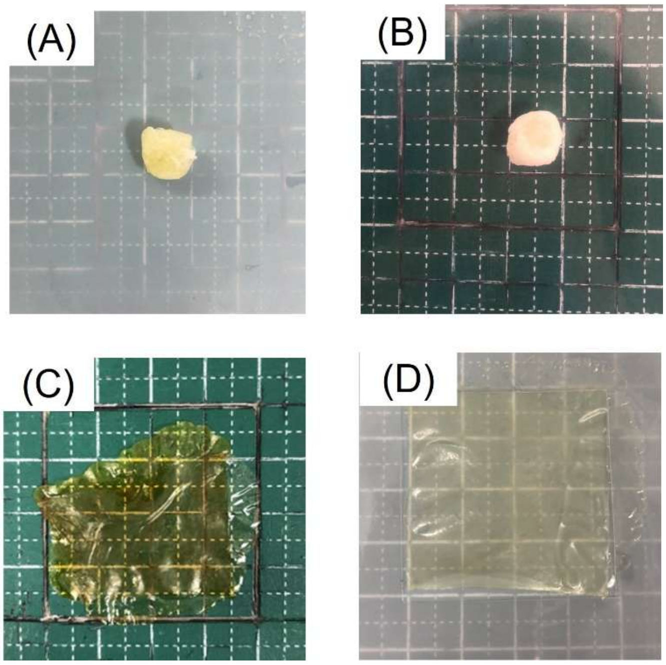

Fig. S6. Photographs of (A) a CS/CD-CHI gel, (B) a CS/CHI gel, (C) a CS/CD-CHI film, and (D) a CS/CHI film. These films were hot-pressed at 120 °C.

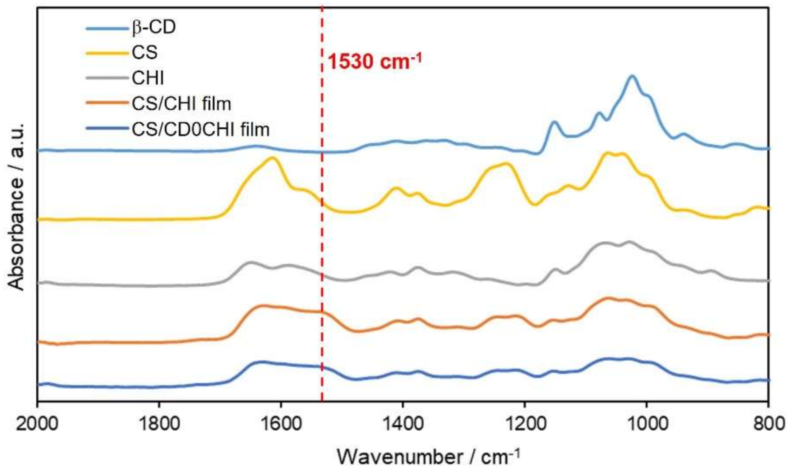

Fig. S7. FT-IR spectra of β-CD, CS, CHI, CS/CHI films, and CS/CD-CHI films.

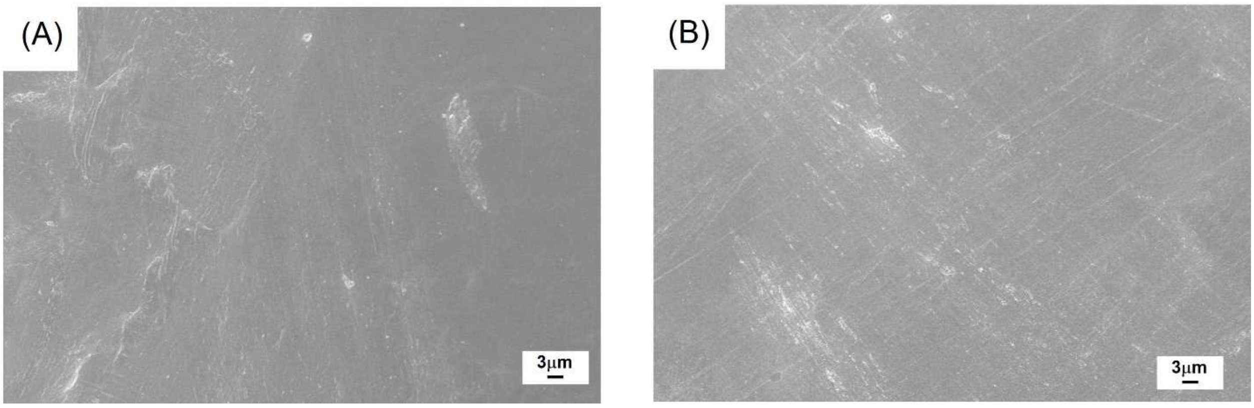

Fig. S8. SEM images of (A) CS/CD-CHI films and (B) CS/CHI films. The magnification ratio was 1000.

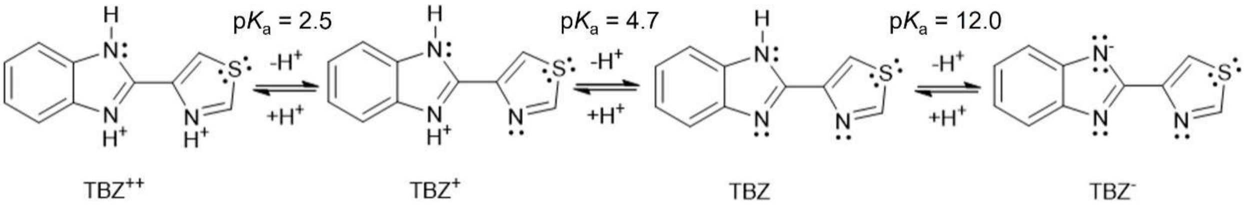

Fig. S9. Ionization of TBZ[4,5]

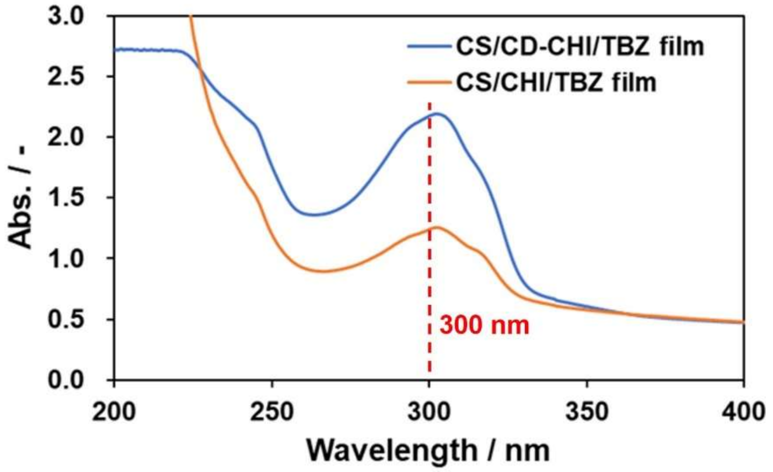

Fig. S10. UV-Vis spectra of CS/CD-CHI/TBZ film and CS/CHI/TBZ film.

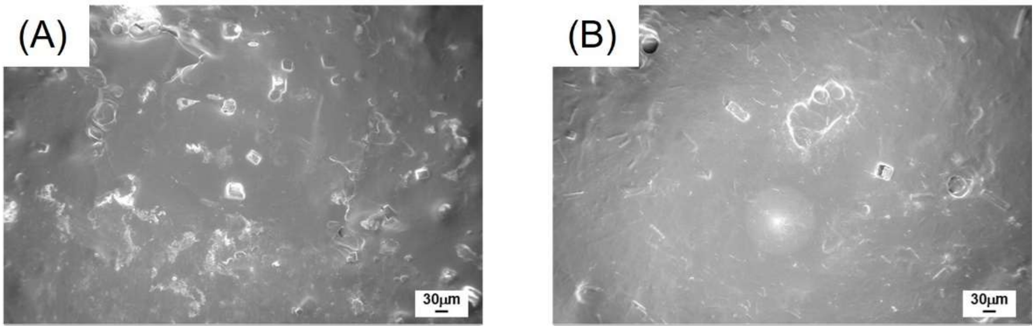

Fig. S11. SEM images of (A) CS/CD-CHI/TBZ films and (B) CS/CHI/TBZ films.

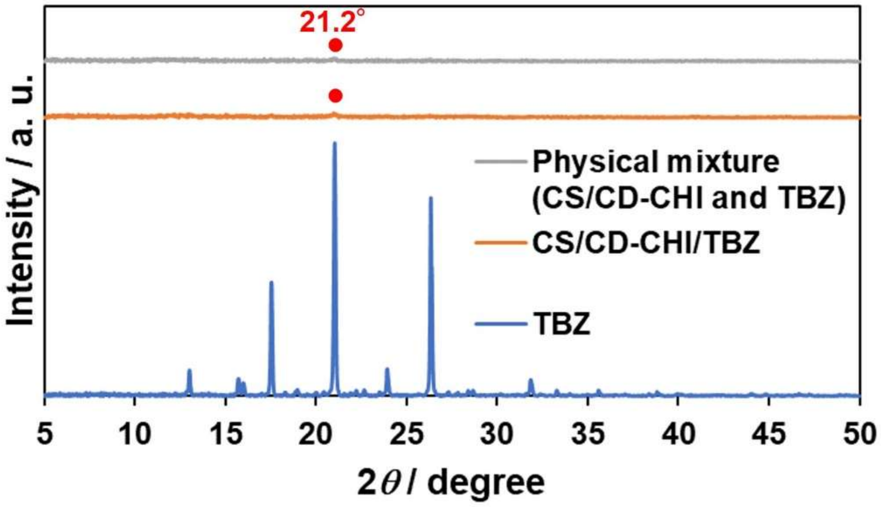

Fig. S12. XRD patterns of CS/CD-CHI film and TBZ (physical mixture), CS/CD-CHI/TBZ film, and TBZ.

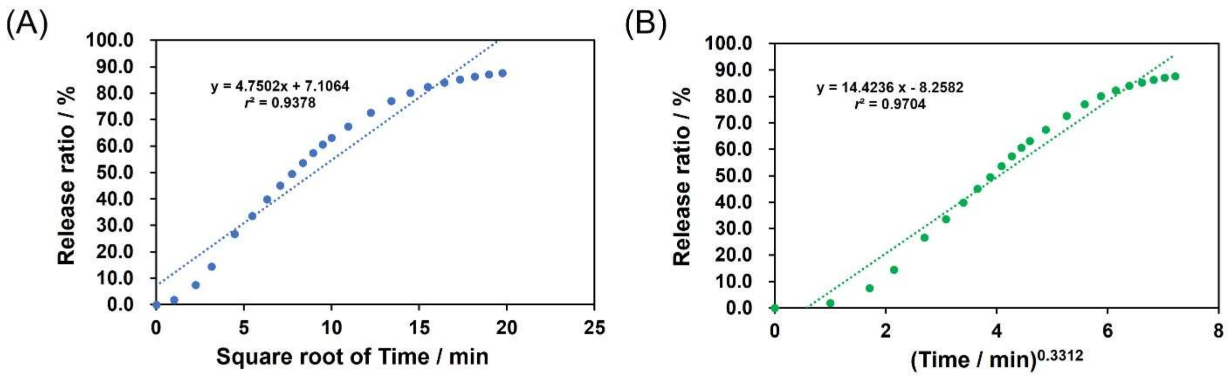

Fig. S13. Analyses of TBZ release profiles from CS/CD-CHI/TBZ films in PBS (pH 7.4) with (A) Higuchi (B) Korsmeyer-Peppas models.

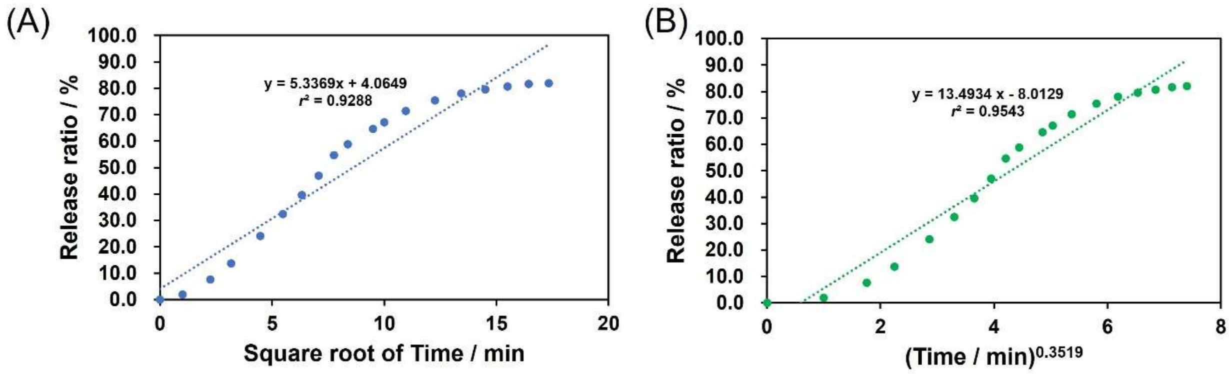

Fig. S14. Analyses of TBZ release profiles from CS/CHI/TBZ films in PBS (pH 7.4) with (A) Higuchi (B) Korsmeyer-Peppas models.

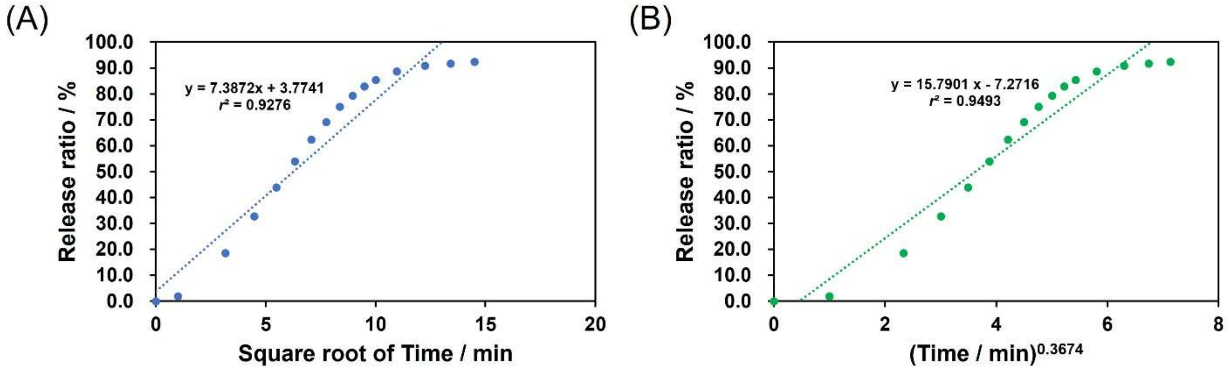

Fig. S15. Analyses of TBZ release profiles from CS/CD-CHI/TBZ films in PBS:EtOH with (A) Higuchi (B) Korsmeyer-Peppas models.

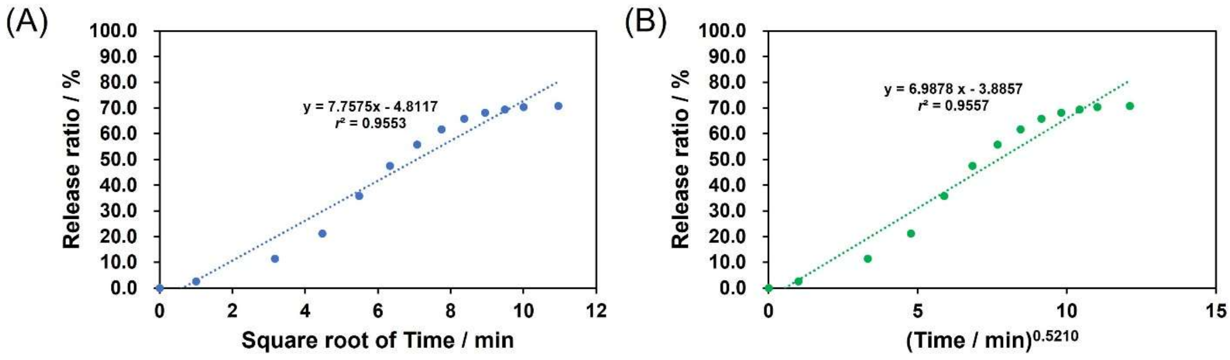

Fig. S16. Analyses of TBZ release profiles from CS/CHI/TBZ films in PBS:EtOH with (A) Higuchi (B) Korsmeyer-Peppas models.

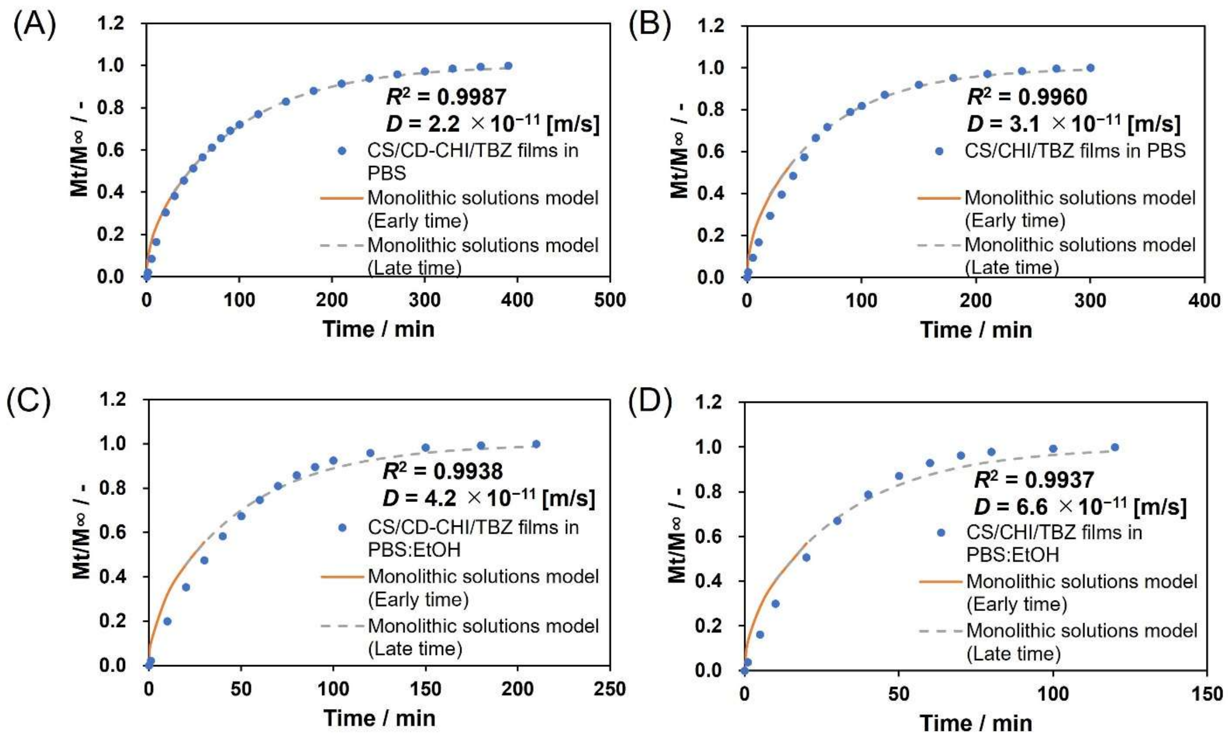

Fig. S17. Fitting for release behaviour of TBZ by the monolithic solutions model using (A) CS/CD-CHI/TBZ films and (B) CS/CHI/TBZ films in PBS, and (C) CS/CD-CHI/TBZ films and (D) CS/CHI/TBZ films in PBS:EtOH. The early time (orange line) was fitted in the range from 0 to 0.6, and the late time (gray dashed line) was fitted in the range from 0.4 to 1.0.

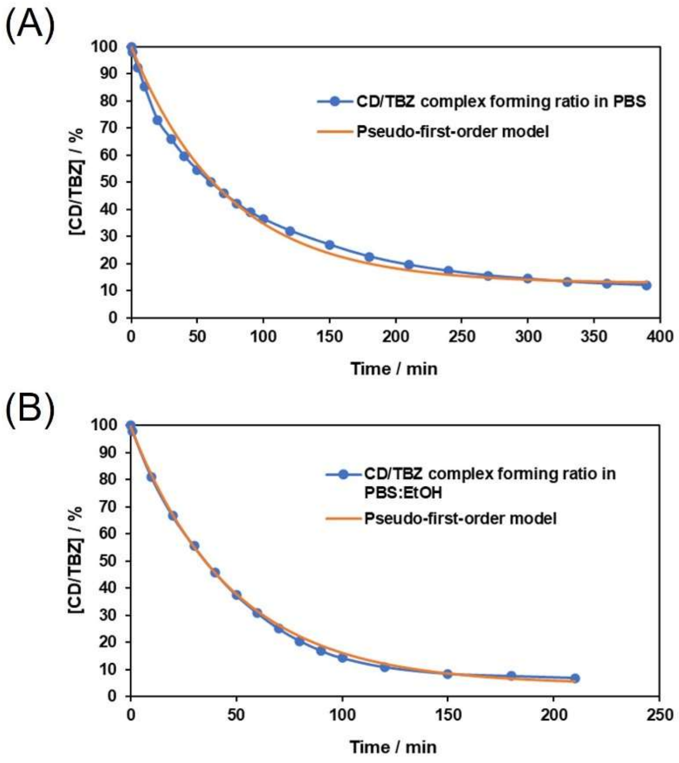

Fig. S18. Curve-fitting of the formation and dissociation reaction of CD/TBZ inclusion complex in (A) PBS or (B) PBS:EtOH (7:3(v/v))) by eq. (13).

REFERENCES

(1) I. C. Salazar, J. L. Mendoza, Y. L. Franco, C. P. Covas, J. Hernández, F. M. Goycoolea, W. A. Monal, Synthesis of regioselective chitosan copolymers with β-cyclodextrin and poly(*N*-isopropyl acrylamide). *J. Polym. Res.*, **2020**, *27*, 112.

(2) M. J. Cornwell, J. B. Huff, C. A Bieniarz, One-Step Synthesis of Cyclodextrin Monoaldehydes. *Tetrahedron Lett.*, **1995**, *36*, 8371–8374.

(3) N. Paiboon, S. Phunpee, U. R. Ruktanonchai, S. Surassmo, R. Methaapanon, T. Vacharanukrauh, S. Assabumrungrat, A. Soottitantawat, Synthesis of cyclodextrin-grafted chitosan: from laboratory scale to pilot scale. *Org. Process Res. Dev.*, **2023**, *27*, 2309−2316.

(4) G. R. S. Cavalcanti, M. G. Fonseca, E. C. S. Filho, M. Jaber, Thiabendazole/bentonites hybrids as controlled release systems. *Colloids Surf. B*, **2019**, *176*, 249−255.

(5) O. F. O. Neto, A. Y. Arenas, A. H. Fostier, Sorption of thiabendazole in sub-tropical Brazilian soils. *Environ. Sci. Pollut. Res.*, **2017**, *24*, 16503−16512.
